# *Staphylococcus* species infected by a bacteriophage with a tail that is both curved and contractile

**DOI:** 10.1128/mbio.03829-25

**Published:** 2026-02-06

**Authors:** Sabrina Suhani, Yan Li, Laura Perlaza-Jiménez, Denis Korneev, Cara Press, Tze Y. Thung, Han-Chung Lee, Joshua J. Iszatt, Ralf B. Schittenhelm, Christopher J. Stubenrauch, Rhys A. Dunstan, Joshua M. Hardy, Anthony Kicic, Trevor Lithgow

**Affiliations:** 1 Infection Program, Biomedicine Discovery Institute and Department of Microbiology, Monash University 214149, Clayton, Australia; 2 Monash Genomics and Bioinformatics Platform, Monash University, Clayton, Australia; 3 Ramaciotti Centre for Cryo-EM, Monash University, Clayton, Australia; 4 Monash Proteomics & Metabolomics Platform, Monash University, Clayton, Australia; 5 Occupation, Environment and Safety, School of Population Health, Curtin University 1649, Perth, Australia; 6 Wal-yan Respiratory Research Centre, the Kids Research Institute Australia, The University of Western Australia 2720, Perth, Australia; 7 New Medicines and Diagnostics Division, Walter and Eliza Hall Institute of Medical Research 548496 , Parkville, Victoria, Australia; 8 Department of Medical Biology, The University of Melbourne 2281, Parkville, Victoria, Australia; 9 Department of Respiratory and Sleep Medicine, Perth Children's Hospital 60081, Nedlands, Australia; 10 Centre for Cell Therapy and Regenerative Medicine, School of Medicine and Pharmacology, The University of Western Australia and Harry Perkins Institute of Medical Research 2720, Perth, Australia; University of Pittsburgh, Pittsburgh, Pennsylvania, USA

**Keywords:** virion morphology, structural proteins, MRSA, phage therapy

## Abstract

Using a selective plating strategy for staphylococci, we surveyed the local community wastewater and purified 16 independent isolates representing the following seven species of *Staphylococcus: S. cohnii*, *S. equorum*, *S. lentus*, *S. nepalensis*, *S. sciuri, S. shinii,* and *S. xylosus*. *Staphylococcus aureus* was not detected. The wastewater also served as a source to identify a bacteriophage (phage), referred to here as JS1, that could infect all these species of *Staphylococcus*, as well as a range of clinical *S. aureus* strains, including methicillin-resistant isolates. The class *Caudoviricetes* are tailed phages, and classification systems recognize the following three major morphotypes: the *Myo-*like (medium-to-long, straight, contractile tails), *Sipho-*like (long, flexible, non-contractile tails), and *Podo-*like (very short, rigid tails). Electron microscopy showed that JS1 virions have 252 nm long, curved, contractile tails. Curvature analysis showed that this represented a range with a 1/R value of 7.6 ± 1.3 μm^−1^, where R is the radius of curvature. Phage JS1 also encodes hydrolases that are assembled onto the phage virions. One of these hydrolases, JS1_0224, was biochemically characterized and found to etch regions from the Staphylococcal cell wall. The possibility that these on-board hydrolases and the curvature of the long contractile tails are advantageous to the phage for navigating through the cell wall of these various species of *Staphylococcus* is discussed.

**IMPORTANCE:**

Past work has seen over-representation of *Staphylococcus aureus* clinical isolates in genome and biology studies on staphylococci. Here, we show by a selective plating analysis of municipal wastewater that independent isolates representing seven other species of *Staphylococcus* were recovered (*S. cohnii*, *S. equorum*, *S. lentus*, *S. nepalensis*, *S. sciuri, S. shinii,* and *S. xylosus*), as readily identified in the samples. Genome sequence analysis revealed some species-specific antibiotic resistance profiles across the strains, and a bacteriophage was isolated that had a cross-species host range. Using this broad biological approach to analyze staphylococci has identified a phage with a broad killing range, and this phage is morphologically distinct from the three known types of tailed phages.

## INTRODUCTION

The genus *Staphylococcus* contains around 60 species and subspecies of gram-positive bacteria, which have been found to colonize a variety of environments from fermented foods (1–3) to animals, including humans. In terms of niches on the human body, skin and mucous membrane are preferred by coagulase-negative (CoNS) staphylococci, whereas additional sites such as sweat glands, ear canals, the pharynx, axilla, and perineum can be colonized by coagulase-positive (CoPS) *Staphylococcus* (4, 5). Most studies (6) have focused on clinical isolates of the species *Staphylococcus aureus* because it is one of the primary sources of skin and soft tissue infections in humans (7–9); one such study showed that as few as 10% of skin and soft tissue infections are caused by staphylococcal species other than *S. aureus* (10). Skin and soft tissue infections also serve as an entry point for staphylococci into the blood (causing sepsis) (11, 12) or bones (causing osteomyelitis) (13, 14). In Australia, there are specific concerns regarding skin and soft tissue infections caused by staphylococci because Aboriginal children suffer these more than children anywhere in the world (15, 16). Children in indigenous communities in Australia are disproportionately affected by sepsis (three times more than non-Aboriginal children) and osteomyelitis (10 times more than for non-Aboriginal children) (14, 17, 18).

Extensive use of the β-lactam antibiotic methicillin to treat *S. aureus* infections is thought to be the driver for the evolution of methicillin-resistant *Staphylococcus aureus* (MRSA). These strains are often resistant to other antibiotic classes too (19), with recent concerning evidence of the simple mechanisms by which MRSA is evolving to be resistant to last-line antibiotics such as linezolid (20–22). Many of these mechanisms for antibiotic resistance would be readily transferable into species other than *S. aureus,* and this could further impact human health. Based on trials with the use of bacteriophages (phages) to treat MRSA infections, there are calls to develop phage therapeutics against multiple species of *Staphylococcus* (23–30). It has been argued that moving beyond the current limited knowledge of *Staphylococcus* phage host interactions will require studying phage-host biology with species other than *S. aureus* (6).

Tailed bacteriophages belong to the class *Caudoviricetes* (31) and must penetrate the bacterial cell envelope to inject their genomic DNA. The primary function of the tail is to mediate both processes: envelope binding and gated DNA release. The phage tail is a complex, multiprotein structure that mediates attachment, digestion, and penetration of the cell wall (32, 33). Previous classification systems used families based on the following three major morphotypes of tailed phages: *Myoviridae*, *Siphoviridae,* and *Podoviridae*. However, genomic diversity and complexity have made it clear that the three morphology-based families require re-evaluation using more rigorous, sequence-based classification (31, 34). Establishing new, sequence-based families for the tailed phages is ongoing, with consensus already on discrete families of contractile phages (Ackermannviridae, Chaseviridae, and Herelleviridae [35–37]), families of non-contractile long-tailed phages (Demerecviridae and Drexlerviridae [36]), as well as a new family of the short-tailed (podo) phages (Autographiviridae [36]). Nonetheless, the characteristic features of three morphotypes remain such that myophages have medium-to-long, straight, contractile tails; siphophages instead have long, flexible, non-contractile tails; and podophages have short, rigid tails. The long, flexible tail tubes of phages, such as T5, λ, and χ, with characteristic siphophage morphology, lack any outer sheath and therefore cannot contract. Several cryo-electron microscopy-based studies have revealed the means by which the long flexible tubes are assembled and how they maintain structural integrity despite their extreme flexibility (38, 39). Myophage morphology is characterized by phage T4, which has a rigid inner tail tube surrounded by an outer contractile sheath. Myophage tails terminate in a baseplate complex displaying tail fibers and/or tail spikes and a central tail spike, all of which can interact with the bacterial surface to thereby trigger contraction (40, 41).

As part of an ongoing research program to detect and characterize phages of unusual morphology, we sought to study phage-host biology with staphylococcal species other than *S. aureus*. We assessed local community wastewater using a selective plating strategy and purified 16 independent isolates representing seven species of *Staphylococcus: S. cohnii*, *S. equorum*, *S. lentus*, *S. nepalensis*, *S. sciuri, S. shinii,* and *S. xylosus*. Antibiotic resistance markers were found as a species-specific difference, such as the methicillin resistance from *mecA*/*mecA1* in all four isolates of *S. sciuri*. At three different sampling sites, independent isolates of *S. xylosus* were discovered, and one of these was used as a host to identify an endemic phage. This phage, JS1, was found to infect all species isolated here as well as a range of *S. aureus* (including MRSA) clinical isolates. DNA sequencing and cryo-electron microscopy revealed that the genome of phage JS1 is large (143 kb) and is packaged into a large icosahedral capsid 92 nm in diameter. The virions were predicted to be composed of 29 protein components. These predictions were validated experimentally by applying mass spectrometry to the purified phage virions. Further evaluation of JS1 virion morphology revealed it to conform to neither the classic myophage morphology (medium-to-long, straight, contractile tails) nor siphophage morphology (long, flexible, non-contractile tails). Instead, JS1 virions have tails with a range of curvatures that can also contract. AlphaFold 3 models showed how the curvature in a phage tail tube requires loose inter-ring connections that can stretch on one side and compress on the other to allow flexion of the tube without breaking. To determine whether this is a feature of phage JS1 that was important in an infection context, we used electron microscopy to map the thickness of the staphylococcal cell wall and determine how deeply the phage penetrates this host cell wall. The 219 nm phage tails appear to completely penetrate the cell wall, which, at its thickest points, is only 70.88 nm, requiring that the phage enter tangentially and/or in curved pathways through the wall to reach the underlying cell surface.

## RESULTS

### Isolation and characterization of *Staphylococcus* from wastewater samples

**Fig 1:**
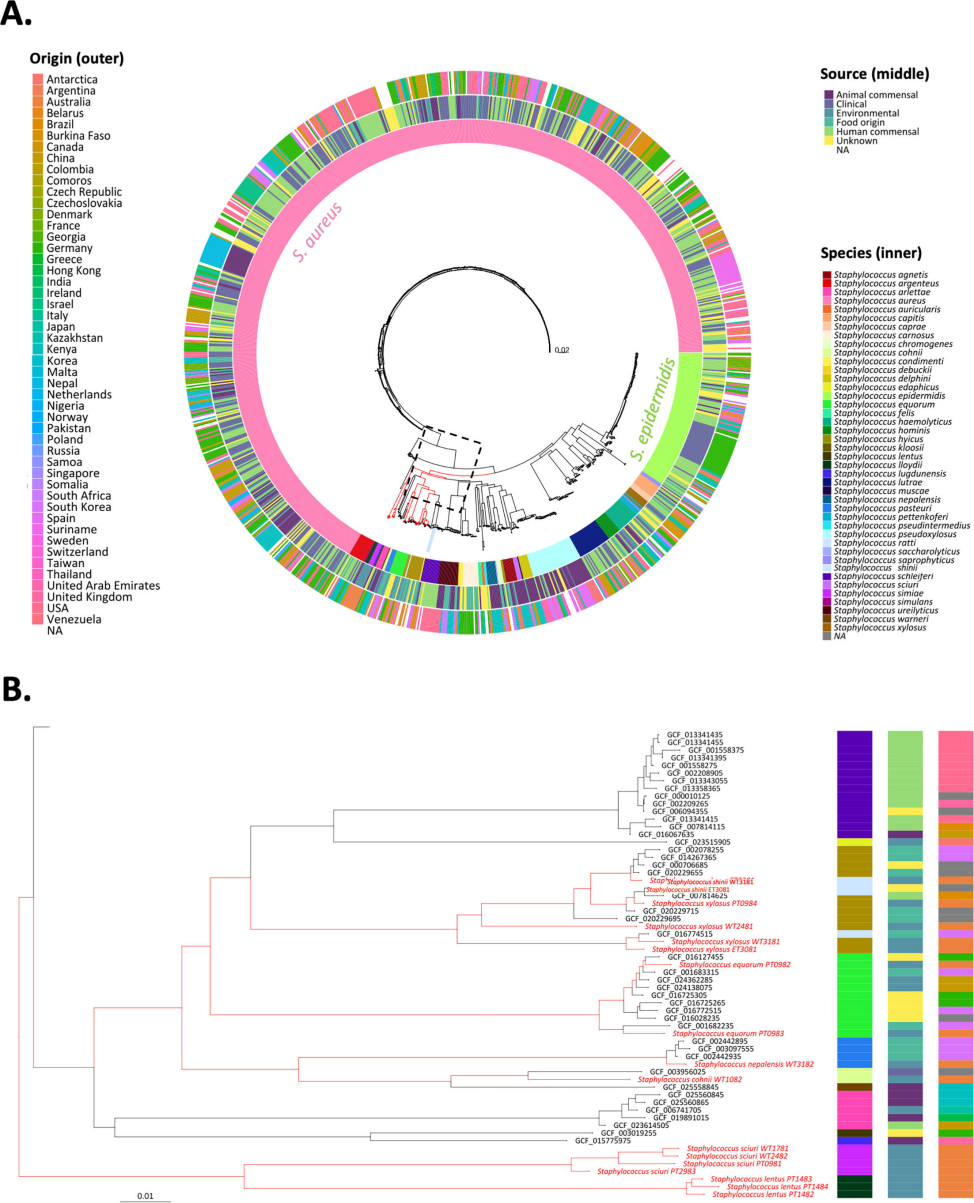
Genome features that differentiate the staphylococcal species. (**A**) Phylogenetic tree of 1,176 *Staphylococcus* strains with complete genome data and the 16 additional strains isolated in this study (branches highlighted in red). Phylogenetic relationships were inferred using Mash distances, calculated with the MASHTREE tool. Branch lengths were scaled according to the Mash distances, as indicated by the scale bar. Annotated alongside the tree are elements of the associated metadata where available (Table S2) encompassing the physical sourcing of the samples (Source, middle ring) and country of origin (outer ring), as well as the taxonomic classification (Species, inner ring). The over-representation of *S. aureus* (pink) and *S. epidermidis* (green) is highlighted in the inner ring. Note, the box with dotted lines has been expanded to show details for the most closely related strains in panel **B**. (**B**) Focused segment of the phylogenetic tree from the 16 *Staphylococcus* strains sequenced and assembled in this study is indicated (branches highlighted in red). The related sequences from NCBI are designated with accession ID codes. Phylogenetic relationships were inferred using mash distances calculated with MASHTREE, based on whole-genome sequences. Branch lengths are proportional to the Mash distances, as indicated by the scale bar, and are consistent with the scaling in the main tree.

*Staphylococcus* can grow on mannitol salt agar (MSA) because they can tolerate the high salt levels in this growth medium, and they can utilize mannitol as a carbon source. Wastewater samples spread on MSA plates resulted in several colonies of bacteria where mannitol fermentation lowered the pH of the media, such that the pH indicator (phenol red) turned yellow in halos around the yellow-colored colonies. Purification, isolation, and whole genome sequence analysis revealed 16 *Staphylococcus* isolates belonging to seven different species (Fig. 1A; Table S1). All seven species isolated in this study are CoNS staphylococci, and the genome sequence identifiers are presented in Table 1. To understand how these strains from local wastewater relate to other sequenced staphylococci from around the world, we established a phylogeny based on the core genome sequences of a total of 1,176 *Staphylococcus* isolates in the NCBI database that have complete genome sequence information (see Materials and Methods and Table S2). Consistent with previous studies (6), our analysis showed *S. aureus* to be grossly over-represented in the data set (Fig. 1A). The genome sequence data were interrogated using the ResFinder tool (42) for the presence of genes that confer resistance to relevant antimicrobial drugs: erythromycin, spiramycin, telithromycin, fosfomycin, clindamycin, tiamulin, and cefoxitin. A multi-drug resistance phenotype was predicted in 12 out of the 16 strains, with a methicillin-resistant phenotype predicted from the presence of the *mecA* gene in 25% of the strains (Table 1).

**TABLE 1:** Characterization of bacteria by predicted drug-sensitivity and phage-resistance testing^*a*^

Bacterial strain	Genome accession ID	Predicted resistance phenotype	Predicted resistance gene	Resistance to phage JS1
*S. nepalensis* WT3182	CP188082	None	N/A	–
*S. cohnii* WT1082	CP188078	Erythromycin and telithromycin	*Msr*	*–*
*S. equorum* PT0982	CP188077	Erythromycin, spiramycin, and telithromycin	*Mph*	*–*
*S. equorum* PT0983	CP188074	None	N/A	–
*S. lentus* PT1482	CP188073	Erythromycin, spiramycin, and telithromycin	*Mph*	Resistant
*S. lentus* PT1483	CP188069	Clindamycin, erythromycin, spiramycin, and telithromycin	*erm*, *mph*	Resistant
*S. lentus* PT1484	CP188068	Erythromycin, spiramycin, and telithromycin	*Mph*	Resistant
*S. sciuri* PT2983	CP188067	Cefoxitin, clindamycin, and tiamulin	*mecA*, *sal*	Resistant
*S. sciuri* WT1781	CP188065	Cefoxitin, clindamycin, and tiamulin	*mecA*, *sal*	Resistant
*S. sciuri* WT2482	CP188064	Cefoxitin, clindamycin, and tiamulin	*mecA*, *sal*	*–*
*S. sciuri* PT0981	CP188063	Cefoxitin, clindamycin, and tiamulin	*mecA*, *sal*	Resistant
*S. xylosus* PT0984	CP188056	Fosfomycin	*fosD*	*–*
*S. xylosus* WT2481	CP188055	Erythromycin, spiramycin, and telithromycin	*Mph*	*–*
*S. xylosus* ET2381	CP188052	None	N/A	–
*S. shinii* ET3081	CP188208	Fosfomycin, erythromycin, spiramycin, and telithromycin	*fosB4, mph*	*–*
*S. shinii* WT3181	CP188207	Erythromycin, spiramycin, and telithromycin	*Mph*	*–*

^*a*^ AMR phenotypes were predicted with ResFinder, based on the presence of the indicated antimicrobial resistance genes, where N/A denotes no output from the predictor. Phage sensitivity was determined by clearance of bacterial lawns, and – means no clearance spots were observed.

Four of the isolates represent the species *S. sciuri*, three are *S. lentus* and three more are *S. xylosus*; two each of *S. equorum* and *S. shinii* were identified, and there was one isolate each of *S. cohnii* and *S. nepalensis*. Many of the strains collected in this study were seen to cluster together with previously described strains of the same species isolated from food sources. For example, the cluster (Fig. 1B), which encompasses the 14 genome sequences from *S. xylosus* (one of which is designated as *S. pseudoxylosus*), comes from South Korea, Brazil, or Australia. None of these 14 strains were documented as being isolated from clinical samples, instead being isolated from food, wastewater, or unknown sources. Similar findings were made for *S. equorum* and *S. nepalensis*. The cluster (Fig. 1B), which encompasses the 12 genome sequences from *S. equorum,* shows that the two which we contributed from Australia sit in distinct subclusters: *S. equorum* PT0983 with a food isolate GCF_001682235 (*S. equorum* C2014) from South Korea, while *S. equorum* PT0982 clusters together with the remaining nine strains from environmental and food sources in South Korea, China, and Germany. The cluster (Fig. 1B), which encompasses the four genome sequences from *S. nepalensis,* has three food isolates from South Korea, with the fourth being *S. nepalensis* WT3182 isolated from the environment in this study. The observed genomic similarities in strains in food and wastewater prompted us to ask whether cross-reactive phages might be present in the water sample, by isolating phages against the most prevalent hosts and testing such a phage for cross-reactivity with other staphylococcal species.

### Characterization of phage JS1

**Fig 2:**
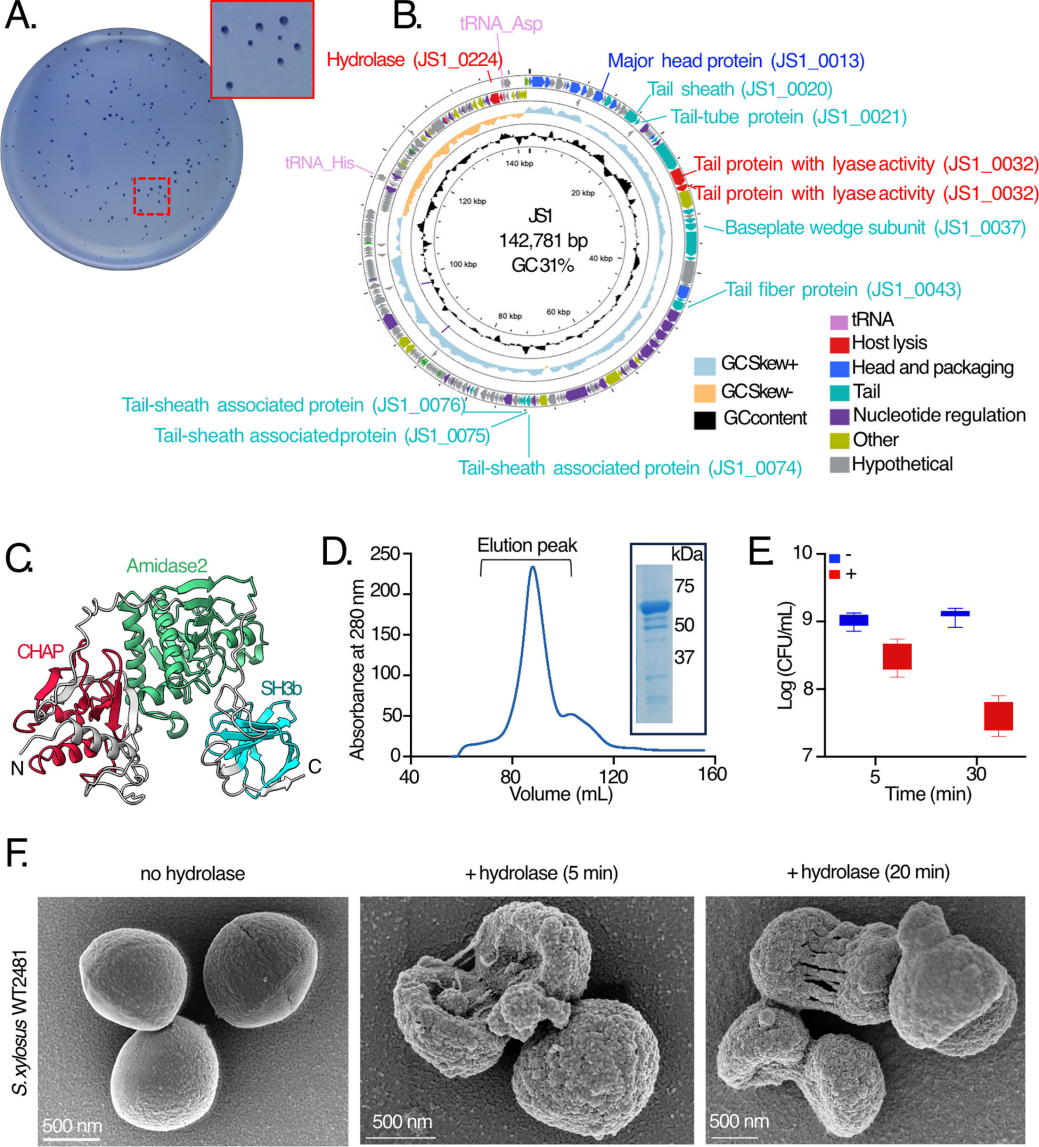
Purification and characterization of *Staphylococcus xylosus* phage JS1*.* (**A**) Plaque morphology of phage JS1. As highlighted in the magnified inset, the plates showed heterogeneous plaque morphology even after five rounds of purification on double-layer agar plates. (**B**) Genome organization of *Staphylococcus* phage JS1 generated using Proksee (45), where the pink arrows in the outermost circle represent tRNAs, and the color-coded arrows represent predicted CDS as indicated in the legend. The inner circle with sky blue and light orange colors represents GC skew, and the black circle inside it represents the GC content. Labeling of a selection of coding sequences provides orientation of the map. (**C**) The protein sequence of the putative hydrolase JS1_0224 modeled by AlphaFold. The ribbon structure is colored according to domain boundaries, from the N-terminal CHAP domain (residues 45–136, crimson), to the central Amidase2 domain (residues 190–387, green) to the C-terminal SH3b domain (residues 409–475, cyan). Details, including the confidence data on the AlphaFold models, are presented in Fig. S2. (**D**) Details of the purification procedure by metal-affinity and size-exclusion chromatography are provided in the Methods section; the size-exclusion profile is shown here. The elution peak represents the fractions that were pooled and assessed for purity by SDS-PAGE and Coomassie blue staining to achieve the final protein preparation. Inset: Coomassie blue-stained SDS-PAGE gel of 13 µg of purified JS1_0224 protein (expected size 56 kDa). Details of the purification procedure by metal-affinity and size-exclusion chromatography are provided in the Methods section. (**E**) A sample of early log-phase *S. xylosus* WT2481 cells (80 µL) was incubated without or with JS1_0224 hydrolase (20 µg), and at the indicated times, samples were withdrawn to assess CFUs on LB agar plates. Nine independent assessments of CFU measurements were made and plotted with the grouped boxplots, where the whiskers indicate the minimum to maximum values obtained. Boxplots in blue represent the values obtained in the absence of hydrolase (-), while those in red represent the data from hydrolase treatment (+). (**F**) Scanning electron microscopy was used to independently assess the activity of the JS1_0224 hydrolase, with samples prepared in its absence (left panel), or with treatment of 20 µg of hydrolase for either 5 min (middle panel) or 20 min (right panel). Representative images are shown. The scale bar represents 500 nm. For one of the panels in this figure, brightness and contrast were adjusted. The original data and this adjusted image are shown in Fig. S5A for comparison.

A phage isolation procedure was established where wastewater samples that had yielded the bacterial isolates were screened for lytic phage. On the host strain *S. xylosus* WT2481, two distinct plaque morphologies (large and small) were observed on the bacterial lawns (Fig. 2A). After five rounds of single plaque purification, both of the phage preparations retained this heterogeneous plaque morphology, which has been observed for Staphylococcal phages in previous studies (43, 44), suggesting that the same phage was isolated twice independently. After purifying each of the phages separately and then sequencing them independently, they were confirmed to be genetically identical. We refer to this phage as JS1. An initial host-range analysis based on spot tests suggested that 10 of the 16 strains were sensitive to phage JS1 (Table 1).

The JS1 genome was found to be large (143 kb) and to encode 241 protein-coding sequences (Table S3). The annotation of the JS1 genome is comparable to other *Staphylococcus* phages from the Family *Herelleviridae* in regard to genes controlling DNA replication and transcription, phage DNA packaging, and host cell lysis (Fig. 2B). The genome of phage JS1 has a 31% GC content overall, with a striking discontinuity when considering the GC skew (Fig. 2B). Approximately 20% of the genome is a segment of higher GC content that includes key bacterial genes, including those encoding a membrane protein, a LysM-type cell wall binding protein, a transposase, and two tRNAs (tRNA^Asp^ and tRNA^His^) that have the features of functional tRNAs (46). The chimeric GC content is seen in *Herelleviridae* phages generally; BLAST analysis using this segment found in the genome sequences of many other phages, which belong to the family *Herelleviridae* (37). Excluding *Herelleviridae* phages from the subsequent analysis meant that no sequences were recovered showing similarity to this segment of phage JS1.

The closest relative to phage JS1 and *Staphylococcus* phage vB_Sau_Clo6, isolated in Argentina against clinic-derived *S. aureus (*47) (Fig. S1A). We recently reported the genome sequence of other staphylococcal phages found in breast milk samples (48) and wastewater (49) in Subiaco, Western Australia. Phages Biyabeda-mokiny 1 (BB1) (48), Biyabeda-mokiny 2 (BB2) (49), and Koomba-kaat 1 (KK1) (49), all belong to the *Herelleviridae* and show overall phylogenetic similarity to phage JS1 (Fig. S1B). However, as its closest known relative, the DNA sequence comparison showed 98% sequence identity between phage JS1 and *Staphylococcus* phage vB_Sau_Clo6 across 89% coverage of the genome length (Fig. S1C). The tools Pharokka (50) and STEP^3^ (51) use distinct evaluations to determine proteins present in phage virions as well as predict their function, and these independent assessments confirmed that the protein components of the JS1 virions are encoded on genes that are not organized into functional units along the JS1 genome (Table 2 and Fig. 2B). To validate the prediction of structural genes, phage JS1 was purified on cesium chloride gradients, and mass spectrometry was used to demonstrate that there are 28 structural proteins in purified JS1 virions (Table 2).

**TABLE 2:** Structural proteins in JS1 virions^*a*^

Gene	Predicted by Pharokka	Predicted by STEP^3^	Pharokka annotation description
JS1_0001	+	-	Terminase small subunit
JS1_0002	+	-	Terminase large subunit
JS1_0003	+	-	Structural protein
JS1_0008	+	-	Portal-associated factor
JS1_0009	+	+	Portal protein
JS1_0011	+	+	Head maturation protease
JS1_0013	+	+	Major head protein (major capsid protein)
JS1_0015	+	+	Tail fiber protein (head-tail connector)
JS1_0016	+	+	Likely tail/neck-related capsid protein
JS1_0020	+	+	Tail sheath
JS1_0021	+	+	Structural protein (tail-tube protein)
JS1_0027	+	-	Structural protein
JS1_0028	+	-	Tail assembly chaperone
JS1_0030	+	+	Structural protein
JS1_0031	+	+	Tail protein with lysin activity (hydrolase)
JS1_0032	+	+	Tail protein with lysin activity (hydrolase)
JS1_0034	+	+	Structural protein (baseplate hub protein)
JS1_0036	+	+	Baseplate protein
JS1_0037	+	+	Baseplate wedge subunit
JS1_0038	+	+	Tail morphogenic protein
JS1_0039	+	+	Baseplate morphogenic protein
JS1_0042	+	+	Likely tail/neck-related capsid protein
JS1_0044	+	+	Tail fiber protein
JS1_0074	+	+	Tail-sheath associated protein
JS1_0075	+	+	Tail-sheath associated protein
JS1_0076	+	+	Tail-sheath associated protein
JS1_0087	+	+	Tail protein
JS1_0208	+	-	Structural protein
JS1_0224	+	+	Hydrolase

^*a*^ The proteins detected by mass spectrometry of JS1 virions are shown with a description of their putative function. For the predictor program evaluation, + indicates those sequences that were predicted to be structural proteins by the software tools Pharokka and STEP^3^, and - indicates those sequences that were not predicted.

In many phages, tail-fiber proteins have hydrolase activity that engages with host cell surfaces (52, 53). Among the proteins identified by mass spectrometry as components of the phage JS1 virion (Table 2), three are protein sequences that are annotated as “lysins” (54): JS1_0031, JS1_0032, and JS1_0224. These three protein sequences generated high-confidence structural predictions, and DALI searches for related structures showed proteins with conserved domains of relevant function, including hydrolases annotated as lysozymes and lyases (Fig. S2). AlphaFold structure models provided support to these relationships, suggesting domain structures characteristic of cell wall hydrolases. JS1_0224 showed structure-based relationships with phage-encoded proteins such as LysK, with a modular structure of two catalytic domains: a cysteine/histidine-dependent amidohydrolase/peptidase (CHAP) domain and amidase-2 (*N*-acetylmuramoyl-l-alanine amidase) domain (Fig. 2C). In addition, the structural model shows a C-terminal SH3b cell wall binding domain (55). While the open-reading frames encoding each of the three candidate hydrolases were cloned into a plasmid for recombinant protein expression, it was only the protein JS1_0224 that was successfully expressed and purified (Fig. 2D). The purified JS1_0224 protein has hydrolytic activity against cell walls, which was supported by two assays. First, the addition of 20 μg of the purified protein to 100 mL cultures showed a clearance of *S. xylosus* by OD_600_ measurement that was quantified to be a ~100-fold reduction in viable colony-forming units (CFU/mL) (Fig. 2E). Second, in an assay where samples of *S. xylosus* WT2481 were treated with the purified JS1_0224 and then analyzed by scanning electron microscopy (SEM). The addition of purified JS1_0224 resulted in pitting and scarring of the cell wall after 5 min of treatment and more severe cell morphology defects, including cell lysis, after 20 min treatment with purified JS1_0224 (Fig. 2F).

### Phage JS1 has a contractile tail sheath

**Fig 3:**
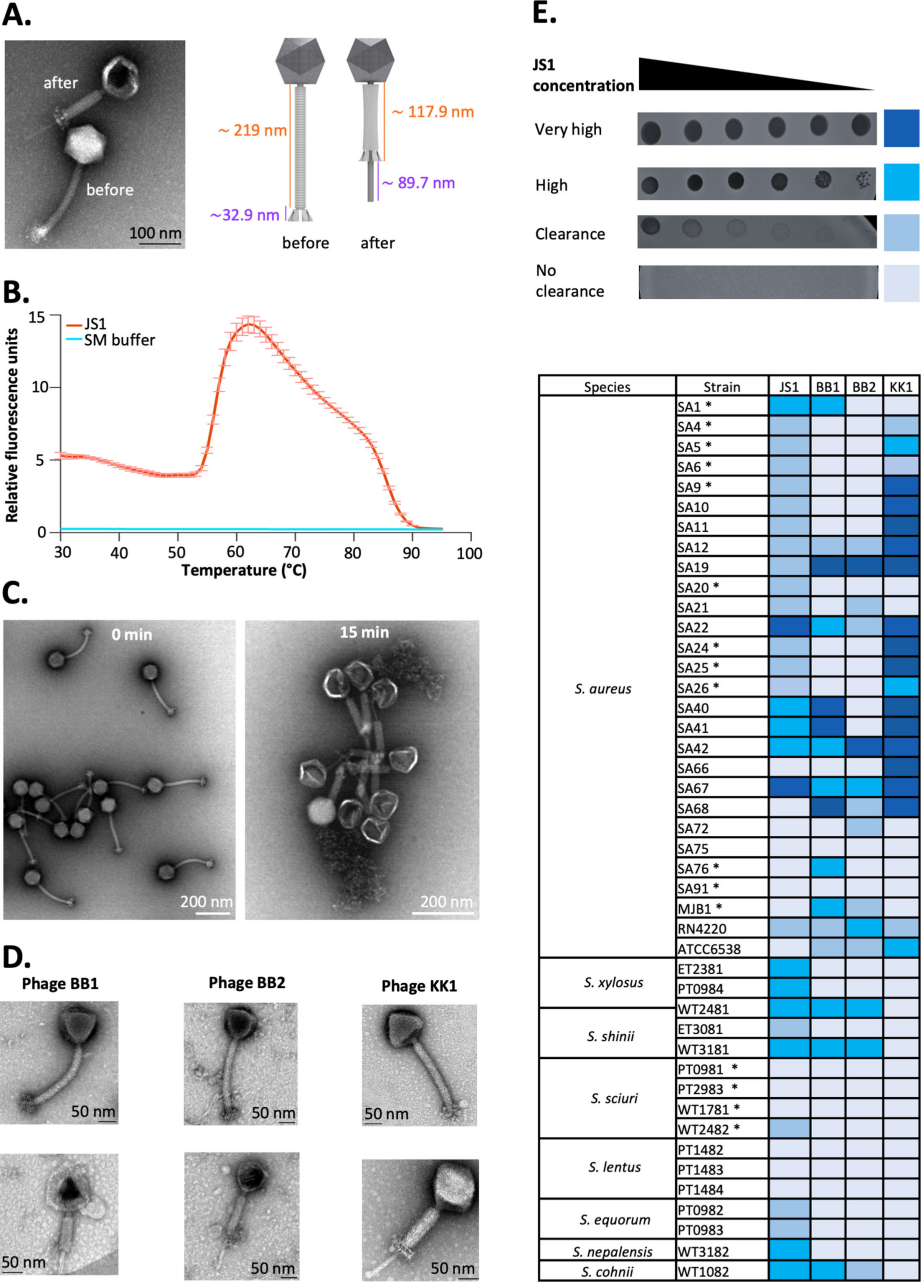
Phage morphology, contraction, and broad host range. (**A**) An example of a contracted phage (after) with a capsid deflated after DNA release alongside an uncontracted phage (before), and a schematic provides the relative measurements before and after contraction. Scale bar denotes 100 nm. The micrographs show the icosahedral capsid (width 91.8 ± 4.2 nm, *n* = 15), tail of length (219.1 ± 8.5 nm, *n* = 15) and tail-tip (length 32.9 ± 1.8 nm, *n* = 15) as a star-shaped structure with multiple spikes. Note in the contracted phage, the negative stain reveals the deflated capsid after DNA has been ejected. (**B**) A phage-stability assay using SYBR Green dye to measure DNA ejection from the phage virions. The phage virions resuspended in SM buffer (Methods) showed that peak fluorescence occurs at temperatures above 60°C (red line). The SM buffer served as a control (blue line) and generated no fluorescence. (**C**) Samples of phage JS1 were prepared either without (20°C) or after a 15 min incubation at 70°C. The micrographs show the icosahedral capsid (width 91.8 ± 4.2 nm, *n* = 15) is intact in the uncontracted phages at 20°C, but it is deflated in the contracted phages after heat treatment. Scale bar denotes 200 nm. (**D**) Transmission electron micrographs of phages Biyabeda-mokiny 1 (BB1), Biyabeda-mokiny 2 (BB2), and Koomba-kaat 1 (KK1). The lower panels show a contracted phage for each of these phages, and the upper panels show the uncontracted virions. Scale bars denote 50 nm. (**E**) The legend shows a sample spot analysis of strains challenged with phage JS1. The dark blue color represents spots that are off-scale for the high-throughput testing and showed equal or better PFU/mL than the isolation host *S. xylosus* WT2481 in plaque assays (Fig. S4). The medium blue color represents spots that show plaques at the highest dilution in the spot testing, equating to 10-fold to 100-fold less PFU/mL than the isolation host *S. xylosus* WT2481 in plaque assays (Fig. S4). The light blue color denotes strains that showed positive spot testing, but only small, hazy plaques at low dilution in plaque assays, and gray represents strains where no positive spot test was detected. In the lower panel, the host-range assessment for the four phages under study: JS1, Biyabeda-mokiny 1 (BB1), Biyabeda-mokiny 2 (BB2), and Koomba-kaat 1 (KK1). The color-coding for their virulence is consistent with the scale shown in the legend. Asterisk indicates methicillin-resistant strains.

Phage JS1 also has a number of proteins that conform to the sequence features of baseplate proteins that are found in contractile phages, including a baseplate protein (JS1_0036), a baseplate morphogenic protein (JS1_0039), and the baseplate wedge protein (JS1_0037) (Table 2). To visualize the morphology of the JS1 virions, transmission electron microscopy (TEM) analysis was used (Fig. 3A; Fig. S3). Screening across the electron micrographs, examples were found of JS1 virions where the tail presented as a contracted form with the outer sheath condensed as a shorter structure revealing the inner tail tube (Fig. 3A). Measurements of these contracted phages (*n* = 15) showed a sheath length of 117.9 ± 8.8 nm, with the resultant extended tube measuring 89.7 ± 6.7 nm. Phage JS1 has an icosahedral capsid (width 91.8 ± 4.2 nm, *n* = 15), the large size of which is consistent with the need to house the 143 kb genome. The tails of phage JS1 are 219.1 ± 8.5 nm long (*n* = 15) with a width 23.7 ± 1.0 nm and were observed to be terminated in a tail tip (length 32.9 ± 1.8 nm, *n* = 15) with a star-shaped structure that has multiple tail spikes emanating from it.

To characterize virion contraction further, a sample of phage JS1 was incubated in a RotorGeneQ thermocycler in a buffer containing SYBR Green, a dye which fluoresces in the presence of DNA. Samples were scanned across a temperature range up to 100°C to monitor for phage contraction, as measured by genomic DNA release. The fluorescence spike was seen above a temperature of 60°C and was largely complete by 80°C (Fig. 3B). With this information, we treated identical preparations of phage JS1 at 70°C for either 0 min or 15 min and then imaged them using TEM (Fig. 3C). As seen previously for phage prepared at 20°C (Fig. 3A), at 0 min of heating, there are a range of straight and curved phage tails, with very few that have contracted. Conversely, the sample heated for 15 min at 70°C showed that 516 of the 518 virions observed had contracted tails and deflated capsids as a result of DNA release (Fig. 3C). These contracted virions are seen to have straightened, such that the 118 nm contracted segment of the virion particles was observed to be straight, and this straight characteristic was noted for a total of 516/516 virion profiles.

Electron microscopy of *Herelleviridae* phages BB1, BB2, and KK1 revealed that they also have contractile tails. While each of the phages is capable of contracting to expose the inner tail tube (Fig. 3D, lower panels), the uncontracted phages show evidence of flexion in the tail (Fig. 3D, upper panels), a feature also seen in the JS1 virions (Fig. 3A and C). Phages BB1, BB2, and KK1 were isolated on clinical isolates of MRSA, but phage JS1 was isolated from wastewater on *S. xylosus*. Thus, we sought to characterize the host range for all four phages.

### Host range analysis

A collection of clinical isolates of methicillin-sensitive *S. aureus* and MRSA was assembled and assayed using a semi-quantitative score (56) based on the titration of phage stock to individual plaques. In this approach, no clearance of the lawn is rated as “no clearance” and colored as gray (Fig. 3E). Some clearance is colored light blue, while the formation of plaques at high titer values is colored medium blue, and clearance zones beyond the dilution tested are colored dark blue. To validate this high-throughput assay in terms of relative plaque-forming activity, the various strains were assessed by standard plating techniques to measure and count individual plaques (Fig. S4). Samples categorized as “dark blue” are equivalent to the plaque-forming activity seen on the isolation host *S. xylosus* WT2481 or greater, while samples categorized as “medium blue” are 10–100 times less active in numbers of plaques formed, but nonetheless, the samples show clearly defined plaques on these strains (Fig. S4).

Several strains were resistant to all four of the phages tested (Fig. 3E); notably, the strains of *S. sciuri* were largely phage-resistant, with only one strain (WT2482) producing plaques in response to one phage (JS1). Comparing the phage-sensitive strains revealed that the four phages had very different host ranges and virulence characteristics. The semi-quantitative score for the efficiency of phage JS1 was determined for the 44 *Staphylococcus* strains across seven species, and phage JS1 was seen to lyse 29 strains. Phage BB1 was also able to infect some strains from all seven species (16 out of 44 strains). Phage BB2 was only able to kill 12 out of 44 strains but was seen to be highly virulent in infecting a different set of the *S. aureus* strains, including the linezolid-resistant *S. aureus* MJB1. Phage KK1 appears to be species-specific, being selective for *S. aureus* (19 out of the 28 *S*. *aureus* strains tested). All of the other species in our collection were resistant to phage KK1.

### The tail tubes of JS1 virions are curved

**Fig 4:**
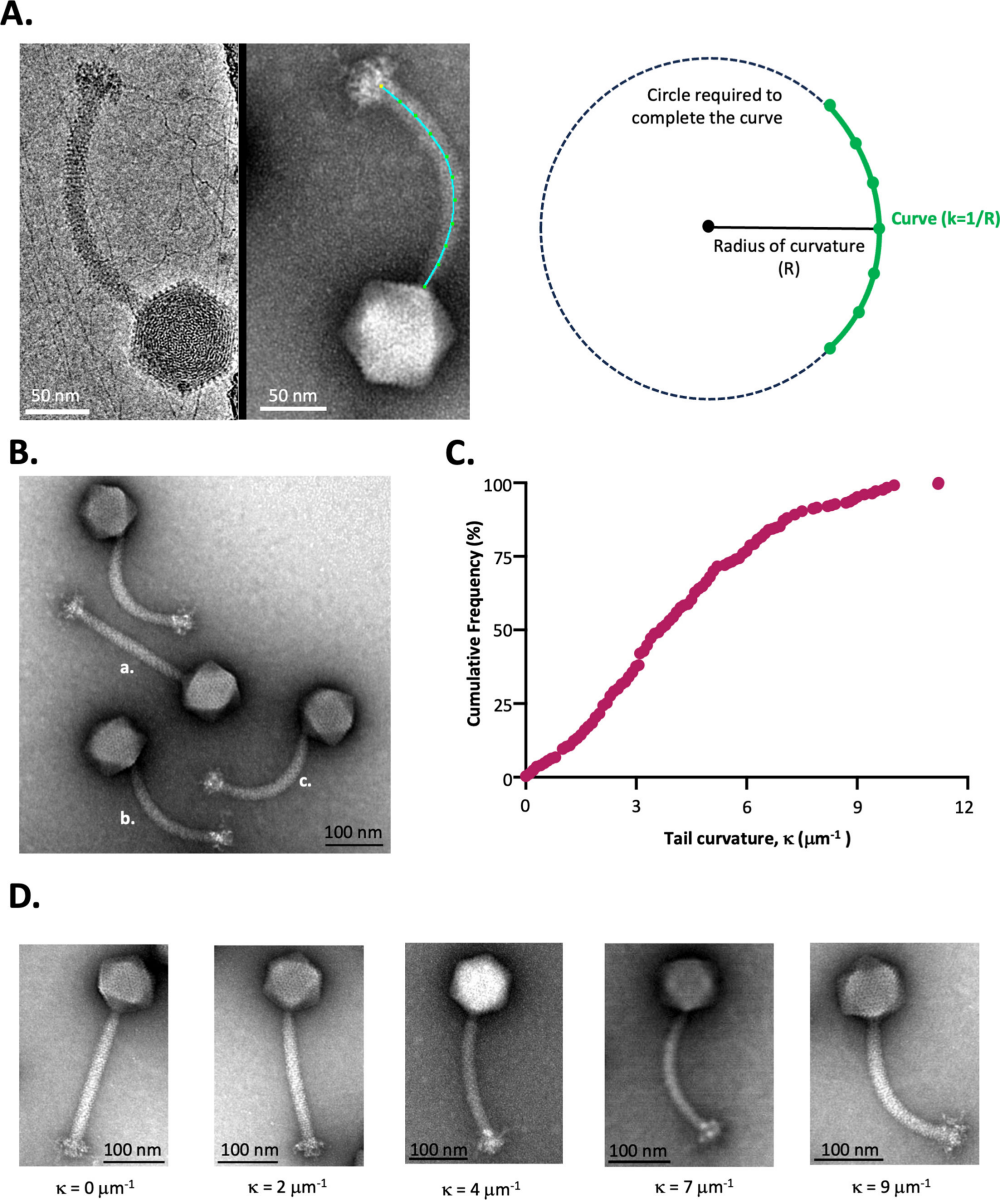
A continuum of tail flexion observed across a population of phage JS1. (**A**) Whether viewed in vitreous ice (left) or by negative-stain enhancement (right), curvature can be seen in the phage JS1 tail. Measurement of phage JS1 curvature using Kappa (see Methods); the user-drawn control points on the image are set by clicking along the length of a curved tail tube as shown in green. Scale bar denotes 50 nm. The schematic shows how this curve is then represented as an arc of a circle of radius I, and the curvature at a point on the line is thereby defined as the inverse of the radius of curvature (1/R). (**B**) Transmission electron micrographs of phage JS1 show the scale and the range of flexion in virion morphology. Broadly, “a” is an example of a tail sitting straight, “b” is an example of a tail slightly curved, and “c” is a tail close to the maximal curvature that can be assumed by phage JS1. Scale bar denotes 100 nm. (**C**) Frequency distribution of virions according to flexion measured from transmission electron micrographs of 250 virions of phage JS1. Data are plotted cumulatively for the 250 virions, to cover the range of κ values that were measured for the 250 virions, with values of the curvature (μm^−1^) plotted on the X axis. For reference, κ values from 0 to 1 represent what appear visually as straight tails. (**D**) Examples of phage JS1 virions with the indicated κ values, representing the straighter tails seen in the κ = 0-1 µm^−1^, the curved tails at either end of the middle group (κ = 2 μm^−1^, and κ = 4 μm^−1^), and the most curved group (κ ≥ 5 μm^−1^). Scale bar denotes 100 nm.

To determine whether the curvatures seen in the phage virions were an artifact of negative staining, a sample of JS1 virions was frozen in vitreous ice and observed by cryo-electron microscopy (Fig. 4A). Similar curvatures were seen in both preparations of phage: in vitreous ice (Fig. 4A, left panel) or after negative staining (Fig. 4A, right panel). For documentation of this morphology, we sought a means to quantify the maximal curvature phage JS1 virions can achieve. Developed for measuring the flexion of microtubules, Kappa (κ) software (57) measures the curvature of a linear structure (as 1/R) with R corresponding to the curvature radius. For example, a straight line would have κ = 0, whereas a circle with a radius of 2 μm has a curve of κ = 0.5 μm^−1^. By selecting 18 virions of extensive curvature, we sought to nominate a maximum value for curvature that JS1 virions can achieve. This curvature of JS1 tails was measured as 7.6 ± 1.3 μm^−1^. The means to quantify maximal curvature was important because it provides a quantitative value that could be useful in comparative analyses with other species of phages. Analysis of the most curved tails of three related phages (Fig. 3D) surveyed 18 virions each and found the curvature radius for the phages to be BB1 (3.9 ± 1.6 μm^−1^), BB2 (4.3 ± 0.9 μm^−1^), and KK1 (2.3 ± 1.0 μm^−1^).

Cursory analysis of electron micrographs of phage JS1 suggested that there is a range of flexions observed across the fixed phage population (Fig. 4B); many tails are observed at lesser than maximal curvatures, and many are seen to be relatively straight (Fig. 4B; Fig. S4). To document this, a total of 250 virions were randomly chosen and assigned to three categories: straight (κ = 0–1), bent (1.1<κ <4.9), and maximally bent (κ>5). The distribution of flexion is graphically depicted (Fig. 4C), and exemplars of the various forms are documented (Fig. 4D).

**Fig 5:**
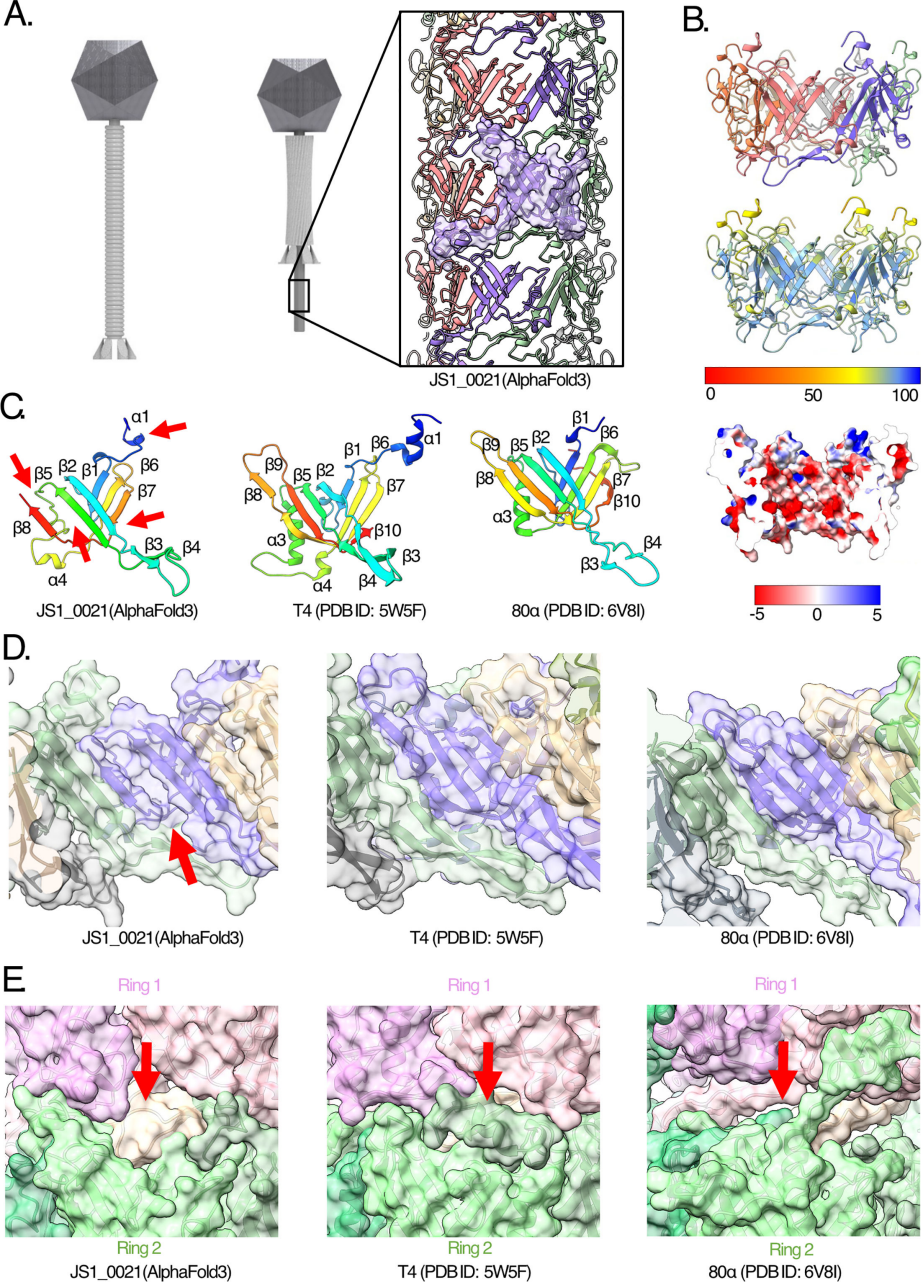
Structural characterization and model for tail-tube flexion in JS1 virions. (**A**) A schematic view of the tail and underlying tail tube in a contractile phage, with an AlphaFold3 prediction for the JS1_0021 tail-tube protein (TTP) assembly. A JS1_0021 monomer is highlighted with a surface representation. (**B**) Upper panel: The AlphaFold3 prediction for the JS1_0021 hexamer colored by individual chain. Middle panel: the same model colored according to the confidence of the prediction (predicted local-distance difference test [pLDDT]) from low (red) to high (blue). Middle panel: the same model colored according to the confidence of the prediction (predicted local-distance difference test [pLDDT]) from low (red) to high (blue). Lower panel: Electrostatic surface rendering of the prediction for the JS1_0021 hexamer shows the negatively charged (red) inner surface for DNA translocation, as previously reported in other phage structures (38, 58, 61–63) (inset) purified JS1 virions were analyzed by SDS-PAGE and Coomassie blue staining, with the four most abundant proteins being excised from the gel and identified by mass spectrometry. (**C**) Detailed view of the TTP subunit for these three phages, viewed from what would be the interior of the tail tube. Red arrows highlight the unpaired β-strands in JS1_21 as well as the poorly formed packing helix, distinguishing JS1_0021 from the TTP subunit of phages T4 (PDB ID: 5W5F) and 80α (PDB ID: 6V8I). In each case, the rainbow color represents the protein from N-terminus (blue) to C-terminus (red), and α-helices and β-strands are labeled in order from N-terminus to C-terminus relative to the T4 TTP. (**D**) Comparison of the JS1_0021 hexamer predicted structure, with the experimental structures of the phage T4 tail-tube hexamer (PDB ID: 5W5F) and phage 80α tail-tube hexamer (PDB ID: 6V8I). An individual tail-tube protein (TTP) subunit is colored purple in each structure for comparison, with secondary structure elements indicated. The red arrow highlights the distinguishing feature in JS1_0021, a groove where there is a missing β-strand. (**E**) The AlphaFold3 model for two stacked rings of the JS1_0021 hexamer: an upper ring (pink) is shown stacked atop a lower ring (green). In comparison with equivalent regions of the structures in phages T4 and 80α, there is a non-complementary surface resulting in gaps between rings of both the 80α and the JS1 tail tube (red arrow).

In both contractile (myo-type) and non-contractile flexible (sipho-type) phages, there is a helical tail-tube assembly formed from stacked hexameric rings of six tail-tube protein (TTP) subunits. In contractile phages, this tail tube is hidden from view by the tail sheath before contraction, being revealed after contraction (Fig. 5A). Structural studies of tail-tube proteins in other types of phages have revealed a conserved fold; this can be seen in both the contractile phages (e.g., T4) and non-contractile flexible tail tubes (e.g., 80α and YSD1) (58, 59). AlphaFold3 (60) modeling confirmed that JS1_0021 is the TTP of JS1, with a hexameric assembly predicted typical of tail-tube assemblies (Fig. 5B). The prediction confidence scores across the model showed a predicted template modeling (pTM) score of 0.73 and an average predicted local-distance difference test (pLDDT) of 82.1. The inner surface of the tail tube is strongly electro-negative, as shown in a cut-away view through the hexamer in Fig. 5B. This observation has been made for other phage tail tubes and is suggested to facilitate ejection of the negatively charged genome from the phage capsid (38, 58, 61–63).

Comparison of an individual JS1_0021 subunit with the TTPs of the T4 contractile phage tail tube (PDB ID: 5W5F) (64), and the non-contractile flexible tail tube of phage 80α (PDB ID: 6V8I) (58) shows that JS1_0021 is predicted to have fewer secondary structure elements and a distinct C-terminal truncation (Fig. 5C). The N-terminal alpha-helix (α1) is shorter relative to T4, and the α3 alpha-helix that normally braces the outside of the tube in other TTPs is disordered (Fig. 5C, red arrows). The C-terminal truncation results in an interruption of the anti-parallel β-sandwich domain with missing β9 and β10 strands in the inner and outer β-sheets, respectively (Fig. 5C, red arrows). Normally, the four inner strands of the β-sandwich in TTPs have hydrogen-bonding patterns between the six subunits such that the inner wall of the tail tube is formed from a continuous 24-stranded β-barrel. In contrast, JS_0021 has a distinct groove between β5 and β8 (Fig. 5D, red arrows). These missing secondary structures may facilitate curvature in the tail tube of phage JS1.

Both in contractile and non-contractile phages, the hexamer is a ring that forms a helical assembly with other hexameric rings to form the overall tail tube. In contractile phages such as T4, there is a highly complementary surface between the rings that maintains their rigidity (Fig. 5E), compared to flexible phages such as phage 80α and YSD1 that have breaks in that complementarity to enable flexion (58, 59). When multiple rings of JS1_0021 were modeled with AlphaFold3, the phage JS1 tail-tube rings show breaks in complementarity more akin to the flexible, non-contractile tail-tube (Fig. 5E).

### Imaging JS1 virions and host cell interactions

**Fig 6:**
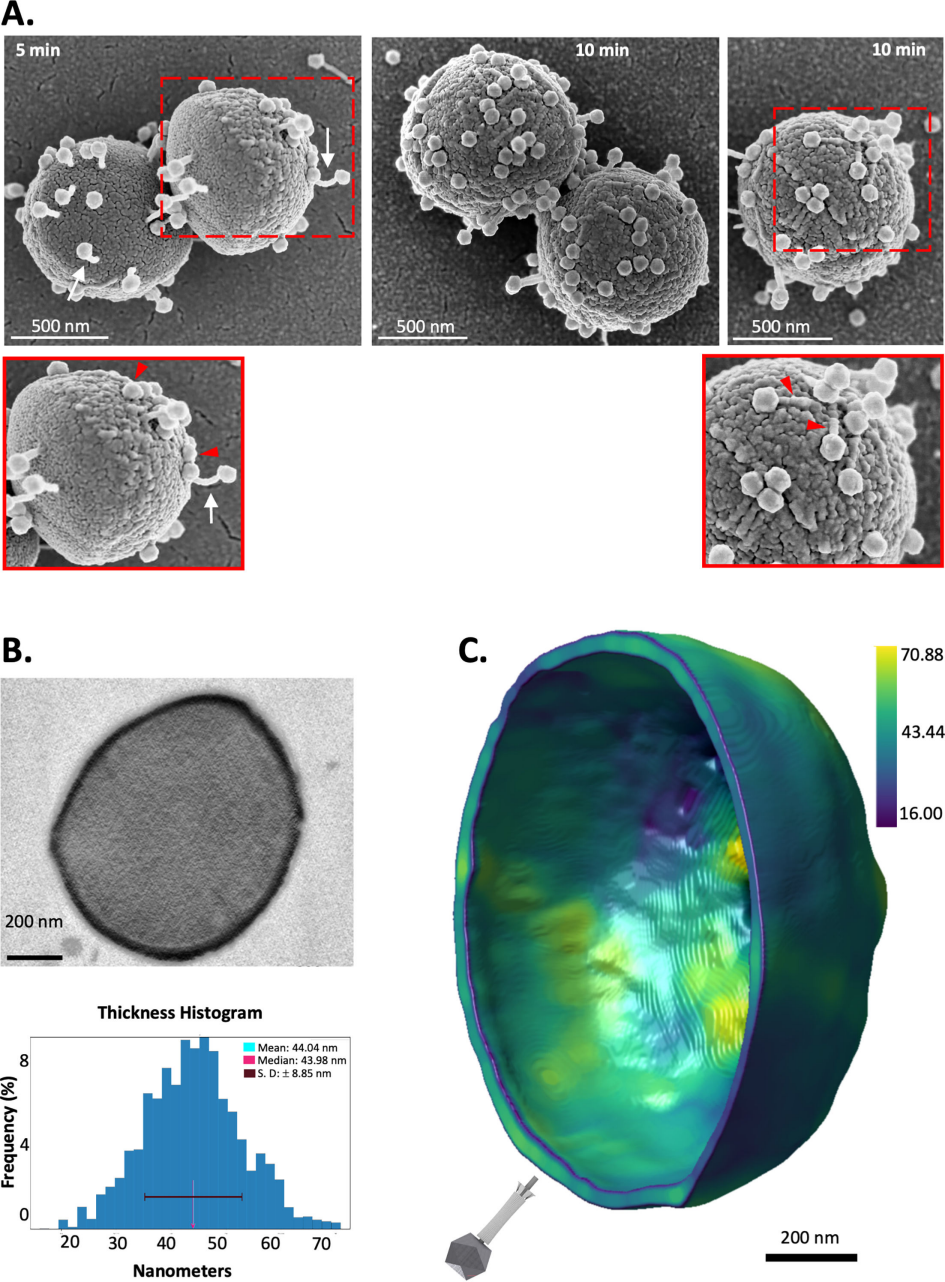
Phage JS1 penetrates deeply into the *S. xylosus* host cell wall. (**A**) Scanning electron microscopy images show virions attached to the surface of the *S. xylosus* host. This phage-host infection experiment (Methods) was established in order to view an early stage of infection where the curved tails attaching to the host cell are marked with white arrows (5 minutes, scale bar 1 μm). Also shown is a later stage of the experiment (10 min, scale bar 1 μm), where more of the ensconced capsids are seen attached to long tubes in or on the cell surface (red arrows). For one of the panels in this figure, brightness and contrast were adjusted. The original data are shown in Fig. S5C for comparison. (**B**) A micrograph of a representative cell profile is shown together with the FIB-SEM data on cell wall thickness plotted as a histogram. The percent frequency of each measurement is plotted against the actual thickness measurement at nanometer resolution. The mean and median measurements are shown. (**C**) A 3D rendering of the cell wall thickness shows the sculpted cell wall features in a spatial context, with the blue-yellow scale denoting thicknesses ranging from 16.09 to 70.88 nm. The average cell wall thickness is calculated for this cell at 43.40 nm. The graphic representation of a JS1 virion is drawn to scale based on the length characteristics measured in Fig. 3A.

Scanning electron microscopy provides a means to visualize phage in association with a host cell and was applied to visualize *S. xylosus* infections with phage JS1. In particular, to determine whether curvature is evident during interactions with the bacterial host. Early in the infection process, in samples fixed within 5 min of applying the phage (Fig. 6A), arrows in the panel at 5 min indicate phages where the tail tube of the infecting phage shows evidence of its curvature. An intriguing observation was that many of the other JS1 capsids in that sample were observed fully ensconced within the host cell surface (Fig. 6A, inset, red arrows). Even more of these ensconced phages were evident in the micrographs taken at 10 min post-infection, and in some cases, structures consistent with the dimensions of the tail tube could be seen on or within the cell surface (Fig. 6A, inset). Given the curvature measured for the tails of phage JS1 virions had revealed that in the uncontracted state, they are capable of considerable flexion, and these data lead us to speculate that the deep penetration into the cell wall could be a combination of the flexion, enabling a meandering path to the cell surface, and the subsequent contraction to a state where some portion of the phage tail has penetrated the *S. xylosus* membrane. In keeping with this idea, we sought to experimentally determine the thickness of the *S. xylosus* cell wall.

The cell wall thickness estimates for *S. aureus* of 20–40 nm (65–68). To evaluate the thickness of *S. xylosus* cell walls, we applied focused ion beam-scanning electron microscopy (FIB-SEM), which reveals the three-dimensional (3D) architecture of the bacterial cells. This technique suggested the *S. xylosus* cell walls have a thickness that ranges depending on where the thickness measurement is taken, with an “average” of 45 nm by FIB-SEM imaging (Fig. 6B). To reconcile this variance of cell wall thickness measurements in spatial terms, the FIB-SEM data were rendered in 3D using Comet Dragonfly software (69) (Fig. 6C). Across the *S. xylosus* cell surface, the cell wall thickness shows regions of up to 70 nm of peptidoglycan and other regions where the cell wall is as thin as 16 nm. A graphical representation of a contracted JS1 virion drawn to scale with this cell wall rendering shows that even after contraction, if the capsid is fully ensconced in the cell wall, it would need to have taken a tangential or meandering path for the phage baseplate to engage the cell membrane below the cell wall.

## DISCUSSION

### Herelleviridae phages JS1, KK1, BB1, and BB2

Félix d'Hérelle co-discovered and first named bacteriophage, and a newly categorized family of phages now bears his name (70). Recent revision of phage taxonomy (71) established the family *Herelleviridae* to include the contractile *Bacillus* phage SPO1, *Staphylococcus* phage Twort, *Staphylococcus* phage K, *Listeria* phage P100, and *Enterococcus* phage φEF24C, all of which infect Firmicutes (37) and do not fit taxonomically with other contractile phages that had been grouped together previously as “Myoviridae” (71). This sequence-based revision meant that more than 90 species of phage have now been placed as a clearly distinct family, but what are their characteristic biochemical and structural features distinguishing them from other phages? According to the current IVS taxonomy report on the phages of *Herelleviridae*, they have isometric, icosahedral capsids of 85–100 nm in diameter and uncontracted tails of 130–185 nm in length. These parameters are also reasonable to describe phage JS1. Phage JS1 has a genome size of 142,781 bp, consistent with the *Herelleviridae* characteristics of double-stranded DNA genomes of 125–170 kbp (37). None of these characteristics, however, distinguishes the *Herelleviridae* virions from other “myovirus morphotype” (contractile) phages. Analysis of phage JS1 revealed a distinguishing feature characteristic of it and three other *Herelleviridae* phages: the tails of JS1, KK1, BB1, and BB2 virions are contractile and curved.

The presence of a contractile tail that is curved was a unique characteristic that we discovered in phage JS1. This morphological feature combines elements of the former group Siphoviridae (long, flexible, non-contractile tails) with elements of the former group Myoviridae (medium to long, non-flexible, contractile tails). While we have not experimentally demonstrated flexibility in these phage tails, the spectrum of curvature seen across the populations of JS1 virions analyzed by electron microscopy, as well as the measured curvature of the tail tubes, is consistent with it being a flexible tail rather than a rigidly curved tail. Structurally, there are specific features in the tail-tube proteins that are required for them to have flexibility (59), and the physics governing the mechanism by which a tail-sheath can contract from a flexible inner tail-tube is currently under study. We cannot rule out an alternative scenario, wherein the JS1 phage virions are assembled with a fixed curvature to the tail locked in place, but that this fixed curvature is distinct for each given virion. Such an alternative would satisfy the data presented here, but it raises the question of how a new locking mechanism such as this could ensure the distinct curvature observed for each virion. While it had gone unnoticed until now, cursory analysis of the literature suggested curvature in the tails of other related phages such as Team1, SAVM03, SAVM04, and SAVM05 (72, 73). Also, in a seminal study on the structure of the Twort-like phage phi812, the supplementary data show curvature in the phage tails after docking onto *S. aureus* host membranes (74). Whether the curved and contractile tails are a defining feature of the *Herelleviridae* will require future efforts to view a broader diversity of this fascinating family of phages.

### Tail tubes that are curved and contractile

In contractile (myo-type) phages and in non-contractile flexible (sipho-type) phages, there is a tail tube formed from a concentric ring of six tail-tube protein subunits. The length of the tail tube is determined by the number of concentric rings that are stacked atop each other; this tail-tube length is exact for any given species of phage but varies between different phages considerably. For example, the contractile phages T4, S-CREM2, and P1 have tail tubes that are, respectively, 92, 245, and 418 nm. All seem to be without curvature (75–77). For non-contractile phages, λ has a 150 nm tail tube (78, 79), SPP1 has a 186 nm tail tube (80), YSD1 has a 220 nm tail tube (81), and P74-26 has a 1,000 nm tail tube (38, 79, 82). Consistent with these published findings, our data suggest no correlation between the length of tails and the degree of curvature. From the TEM images of phages BB1, BB2, KK1, and JS1, the tail lengths do not simply correlate to the average curvature; the shortest tail tube is seen in phage JS1 (208 nm) with the greatest curvature measured at 9.1 μm^-1^, and the other phages with longer tail tubes show uncorrelated average curvature measurements of tail length 209 nm, curvature 3.4 μm^-1^ (for BB2), tail length 212 nm, curvature 1.8 μm^-1^ (for KK1), and tail length 226 nm, curvature 2.7 μm^-1^ (for BB1). Thus, even with quite similar tail lengths (209–226 nm), the maximum curvature appears to be an independent characteristic determined by some other structural feature of the tail tube.

Given that phage JS1 was capable of the maximal curvature measured, it was of interest to compare it to the inter-ring interactions in the tail tubes of contractile phages like T4 can be made with those of non-contractile phages like SPP1, YSD1, and 80α (59, 83). Overall, structural studies have revealed that the T4 tail tube has extensive inter-ring interactions, preventing any prospect of flexion, but there are far less extensive inter-ring interactions seen in the tail tubes of non-contractile phages like SPP1, YSD1, and 80α (58, 59, 83). These hinges allow inter-ring movements of up to 10 Å between the inner and outer faces of adjoining rings, providing for considerable flexion of the overall tail tube. We observed two structural features in the AlphaFold models for the tail tube of phage JS1 that would be relevant to the observed flexion. First, a β-strand is displaced from the inner β-sheet formed by the tail-tube protein JS1_0021 that would provide six points of compression around the hexameric tail-tube ring. Second, the inter-ring interface is more similar to that of the non-contractile phages like SPP1, YSD1, and 80α than it is to the inflexible contractile phage T4.

The curved tail of phage JS1 suggests a distinct structural basis in the tail tube and contractile sheath, which would presumably come at a cost, given the distinct physical constraints needed for contraction of a curved, and potentially, flexible tail tube. Is there any selective advantage to having this fourth morphotype of phage tail? We do not yet know, and can only speculate that the potential for flexion might serve to provide for meandering movement through the intricate tunnels in the cell wall of the host *Staphylococcus* (67, 84). This would not be a precondition for infectivity, since phages of other morphologies may have other means to traverse the cell wall. The cell wall of *S. aureus* is considered to be thick (85, 86), and we showed here that *S. xylosus* has an even thicker cell wall, but with measurements varying across the cell from 16 nm to 70 nm. When live bacteria are viewed by atomic-force microscopy, the topography of the *S. aureus* cell surface is rugged and complex (67, 87, 88), with surface features mapped with dimensions up to 60 nm across the surface and 23 nm deep. Topologically, some of these are seen to be craters with external surface openings far greater (areas of up to 900 nm^2^) than at the floor of these pits where the surface area can be <47 nm^2^ (67).

While speculative, a model in which curved tails may assist the phage to meander through cell wall features is consistent with the images captured of phage JS1 in this study and with images in a previous study of the *Herelleviridae* phage phi812 (73). In terms of staphylococcal cell function, the large cell wall cavities have been suggested to permit efficient passage of nutrients through the dense peptidoglycan (67, 84, 87). Perhaps these irregular cavities also assist phages in wending their way through, prior to engaging with a membrane-located receptor. The cell wall pores do not continue all the way to the membrane, which is thought to be adaptive in that permeation of molecules is assisted without compromising the load-bearing function of the wall in structurally resisting the turgor pressure that would otherwise lyse bacterial cells (67, 84, 87). For this last part of the cell wall passage, hydrolases such as JS1_0224 on the phage virions may be important in accessing a membrane-located receptor to trigger contraction of the tail and ejection of the phage DNA.

### Under-sampling of *Staphylococcus* and staphylococcal phages

In the past, genome sequence analysis of *S. xylosus* focused on its role in the food industry with strains isolated from fermented ham, salami, and kulen, where *S. xylosus* represents a dominant bacterial species (1, 89–98). Throughout the production of these meats, brines introduce *S. xylosus* and other staphylococci (1), and a study of Iberian ham showed that *S. xylosus* was the main species throughout the manufacturing process (99, 100). Our *Staphylococcus* strains were isolated from community wastewater and may be derived from the production and consumption of such foods and/or from washing of commensal skin microbiome constituents into the wastewater flows. We favor the latter as being at least partly the case, given the appearance of other species (*S. sciuri*, *S. lentus*, *S. equorum, S. cohnii,* and *S. nepalensis*) not generally regarded to be food-related bacteria.

The use of related species for phage production is a strategy in the production of phages for biotechnological or medical use, whereby phages can be amplified for purification on related but non-pathogenic species. For example, in the food industry, phages are used to kill *Listeria monocytogenes* but are amplified on non-pathogenic *Listeria innocua (*101–103) with a view that this approach would lead to a safer phage product. Similarly, the use of food-grade bacteria *S. xylosus* has been proposed for propagating phages that target *S. aureus* to avoid the risk of growing a massive volume of pathogenic bacteria and also to reduce the transfer of virulence genes to the phage (72, 104).

The host range of staphylococcal phages was previously regarded as narrow based on distinct, species-specific phage receptors (105), such as the characteristics of cell wall teichoic acids, which show conserved features that distinguish each staphylococcal species (106–108). However, our work with phages JS1, BB1, and BB2 joins other more recent studies that report on staphylococcal phages with broad host range, including the ability to infect diverse species (109–113). In a recent survey of 94 staphylococcal phages, Göller *et al*. showed that some of the broad-range phages have the competence to transduce foreign genetic material from species to species, concluding that staphylococcal phages can be potent vehicles for genetic exchange between host species (113). This also provides a basis on which to potentially design rational strategies for the production of therapeutic phages to kill *S. aureus* and other pathogenic staphylococcal species. Our work adds evidence to the proposal that moving beyond the current limited knowledge of staphylococcal phages will require study of species of *Staphylococcus* other than *S. aureus* (6).

While our characterization of phage JS1 was not directly motivated by potential applications of *Herelleviridae* phages, recent evaluations of phage therapy across major hospitals in Europe underscore the emergence and effectiveness of phage therapy (114, 115), and individual case studies on phage therapy of staphylococcal diabetic foot ulcers showed 6 out of 10 cases phage therapy was judged as having facilitated clinical resolution of infection and limb salvage (30). The *Herelleviridae* family of phages has importance for therapeutic purposes (116–118) with one such phage cocktail called ABSA01 (119) having been shown to be efficacious in the treatment of both diabetic foot ulcers and chronic rhinosinusitis, and the cocktail has been demonstrated to be safe for intravenous administration (120–123). The three phages that are present in the ABSA01 cocktail: Sa83, Sa87, and J-Sa36, are all related to *Staphylococcus* phage K, a founding member of the *Herelleviridae*. As of yet, none of these three phages have been structurally characterized to know if they share the new contractile and curved morphology of phage JS1.

## MATERIALS AND METHODS

### Bacterial strains and growth conditions

Bacterial strains used in this study (Table S1) included MRSA (*S. aureus* SA1 to SA91), MSSA (*S. aureus* SA10 to SA75) strains, *S. aureus* RN4420, S. *aureus* ATCC6538 (Kicic lab, Western Australia), and the methicillin- and linezolid-resistant *S. aureus* MJB1 (Lithgow lab, Melbourne). The species of *Staphylococcus* strains from the Melbourne Water’s three wastewater treatment plants were isolated during this study. All the bacterial strains were stored frozen at −80°C in 50% glycerol. The bacterial strains were seeded on Tryptic soy agar (TSA) (30 g/L tryptic soy broth [TSB] [Edwards Group] with 1.5% agar [Edwards Group]) and grown in TSB overnight (O/N) at 37°C when needed.

The isolated bacterial strains were given unique identifiers where the first two letters of the strain identifiers are the initials for the wastewater treatment plants (PT, ET, and WT for Pakenham Treatment, Eastern Treatment-Bangholme, and Western Treatment-Werribee, respectively). The initials are followed by the date and month of water sampling and the number of positive yellow colonies that were picked from the MSA agar plate for further processing. Thus, *S. equorum* PT0983 shows isolation from wastewater sampling site Pakenham Treatment plant, 098 shows it to be a sample from the 9^th^ of August, and three denotes the third positive colony that was picked from the MSA agar plate.

### Isolation and identification of staphylococci from wastewater

Twenty-one sewage samples were collected from Melbourne Water and South-East Water, via the Environmental and Public Health Microbiology Lab in the Department of Civil Engineering, Monash University, during the period of June 2023 to October 2023. The samples were raw influent after the first screening, which only removes large particles. The samples were from three wastewater treatment plants at Pakenham (11 water samples), Werribee (5 samples), and Bangholme (5 samples). The samples were kept at 4°C until needed.

Mannitol Salt Agar (MSA, Thermo Scientific) plates were used to differentiate colonies by looking for yellow colonies on a halo of surrounding yellow medium, from any other colonies that grew on the plates. At least three mannitol fermentation positive (yellow) colonies from each wastewater sample that showed typical *Staphylococcus* morphology were picked and sub-cultured three times on fresh MSA plates to have clonal isolates for further investigation. The selection of strains relied on criteria such that (i) colonies retained the yellow phenotype after three rounds of plating on MSA, and (ii) where the identification of the isolates as *Staphylococcus* by PCR amplification of a species-specific segment of the gene encoding 16S rRNA (124) genome sequences. All such strains were isolated for a final analysis by whole genome sequencing. Petri dishes containing MSA were seeded with 1 mL of raw sewage samples and incubated at 37°C for 18–24 h (125).

### PCR amplification of 16S rRNA sequences

A thermal cycler (Proflex PCR System) was used for amplification after initial denaturation at 95°C for 10 min, 25 cycles were run of denaturation (95°C for 1 min), annealing (50°C for 1 min), and polymerization (72°C for 1 min) with a final extension time of 72°C for 10 min. Amplified products were evaluated by electrophoresis on 2% agarose gel containing SYBR Safe DNA gel stain (Invitrogen, 0.02 μL/mL), with a fragment of 257 bp expected for the 16S rRNA fragment amplified with these primers (124).

### Genome extraction, sequencing, and analysis of the isolated bacterial strains

For whole genome sequencing, genomic DNA was extracted from overnight cultures (grown on TSB at 37°C) using Genomic-tip 100/G (Qiagen, Inc., United States) according to the manufacturer’s instructions, with the modification that lysostaphin (8 mg/mL; Sigma) was added at the cell lysis step (126). Hybrid genome sequencing (Oxford Nanopore Technologies + Illumina) was performed by Plasmidsaurus (United States) with custom analysis and annotation.

Gene identification using the virulence factor database (VFDB) (127) was carried out for all isolates, and detection of antibiotic resistance genes was performed using ResFinder v4.4.3 (128). The phylogenetic relationship of the newly isolated staphylococcal strains was investigated by genomic, SNP-based analysis using the *Staphylococcus* core genome in Roary v3.12.0. IQ-TREE (129) v2.0 inferred the phylogenetic tree with ModelFinder (130), tree reconstruction, and ultrafast bootstrap (131) (1,000 replicates) analysis. The best-fit substitution model for accurate phylogenetic estimates identified by ModelFinder was GTR+F + R2. The maximum likelihood tree was visualized using iTOL (132) v6.9.

For the global phylogenetic analysis, a total of 1,837 complete genomes from the *Staphylococcus* genus were downloaded from the NCBI database (27 June 2024). In addition, 16 in-house assemblies were generated. Concordant metadata on the country of origin, source of the sample, and species classification were curated from the NCBI database and annotated, resulting in 1,193 strains with complete information. These 1,193 genomes from the *Staphylococcus* genus were subsequently used to generate a phylogenetic tree. The tree was generated using MASHTREE v2.2 (133), a tool designed for rapid phylogenetic analysis based on whole-genome sequence comparisons. Mash sketches were generated using the default parameters, with the option --mindepth 0 applied to include minimum abundance k-mers. The resulting phylogenetic tree was visualized using the R packages ggtree (134) and ggplot2 (135).

### Bacteriophage isolation and purification

For bacteriophage isolation, large particles were collected by centrifugation of the sewage samples (10,000 × *g* for 10 min). The supernatant was filtered through 0.22 µm Millipore sterile filters and supplemented with 5 mM CaCl_2_ (CaCl_2_·2H_2_O, Riedel-de Haen) and MgCl_2_ (MgCl_2_·6H_2_O, Sigma) (136–138). An 800 µL of log-phase *S. xylosus* WT2481 culture was added to the 20 mL of filtered water along with 5 mL of 3× medium (TSB, 5 mM CaCl_2_, and MgCl_2_) and incubated at 37°C, 150 rpm in an orbital shaker overnight. After incubation, the mixture was centrifuged at 10,000 × *g* for 10 min to pellet the bacterial cells, and the supernatant was filtered (0.22 µm Millipore sterile filter) and supplemented with 5 mM CaCl_2_ and MgCl_2_ solution. To screen the filtrate for the presence of bacteriophages, 8 µL of the filtered supernatant was spotted onto plates containing soft agar (0.3% wt/vol TSA) with *S. xylosus* WT2481 and incubated overnight at 37°C. The plate showing a zone of clearing on soft agar was considered positive for lytic phage activity, and the filtrate was used for purification (single plaque isolation) of bacteriophage as described (56). Plaque purification was repeated five times, and the filtrates were stored at 4°C for use in further analysis. High-titer stocks of phage were obtained as previously described (139).

### Phage genome sequencing and analysis

DNA extraction from high-titer phage stocks was performed using DNeasy Blood & Tissue Kit (Qiagen) as previously described (140), and Illumina-whole genome sequencing was performed by Victorian Clinical Genetics Services (VCGS), Australia.

The quality of the raw reads was assessed using FastQC v0.74+galaxy (141), and trimming for low-quality reads was performed using fastp v0.23.2+galaxy0 (142) and BBTools: BBduk v39.01+galaxy0 (143), respectively. The reads were assembled, *de novo*, by Shovill v1.1.0+galaxy2 using SPAdes Genome Assembly Algorithm (144) and were annotated automatically using Pharokka v1.7.1 (49) in combination with PHANOTATE v1.5.1 (145) as the gene predictor and PHROG database (146). Characteristics such as length and GC content of the genome were calculated using Quast v5.2.0+galaxy1 (147). The complete genome sequence was compared with other phage genome sequences using BLASTn from the NCBI (148). Average nucleotide identity between two phages was calculated using FastANI (149). Detection of tRNA was performed using tRNA-Scan (46) (Galaxy Version 0.4), phage lifestyle was predicted using BACPHLIP (150) (Galaxy Version 1.0), and the presence of virulence determinants and antibiotic resistance genes was detected with VFDB, ResFinder v4.4.3, and PhageLeads (151).

Proteomic trees of phage JS1 and other phages (Fig. S3) were generated using ViPTree v4.0 (152) based on genome-wide sequence similarities computed by tBLASTx using Prodigal gene prediction algorithm (153). Nucleotide sequences from any closely related phages from the ViPTree cluster and from BLASTn search were extracted from the NCBI to determine the intergenomic nucleotide sequence similarity. The NCBI accession number of similar phages with other related information is provided (Table S4).

The VICTOR web service (154) was used for the genome-based phylogeny and classification of JS1. All pairwise comparisons of the nucleotide sequences were conducted using the Genome-BLAST Distance Phylogeny (GBDP) method (155) under settings recommended for prokaryotic viruses (156). The resulting intergenomic distances were used to infer a balanced minimum evolution tree with branch support via FASTME, including SPR postprocessing (157). Branch support was inferred from 100 pseudo-bootstrap replicates each. Trees were rooted at the midpoint (158) and visualized with ggtree (159). Taxon boundaries at the species and genus level were estimated with the OPTSIL program (160), the recommended clustering thresholds, and an F value (fraction of links required for cluster fusion) of 0.5 (161).

### Purification of phage virions by cesium chloride gradient centrifugation

An overnight culture of *S. xylosus* WT2481 was diluted 1:100 in 800 mL TSB and grown at 37°C (200 rpm) to reach early stationary phase.

The culture was infected with the high-titer phage lysate and incubated with shaking (200 rpm) at 37°C for 2.5 h. Bacterial cells were then centrifuged (20 min, 11,000 × *g*, 4°C), and the supernatant was filtered (0.45 µm). Sodium chloride was added to a final concentration of 1 M, and phage virions were precipitated from the media by adding Polyethylene glycol (PEG) 8000 (10% final concentration) for incubation overnight at 4°C with gentle stirring. The PEG precipitate was collected by centrifugation (20 min, 11,000 × *g*, 4°C), and the pellets resuspended in SM buffer (100 mM NaCl, 8 mM MgSO_4_, 10 mM Tris pH 7.5) using 1.6 mL/100 mL of precipitated supernatant. An equal volume of chloroform was added to remove residual PEG and cell debris, and the suspension was vortexed for 30 s. The organic and aqueous phases in the solution were separated by centrifugation (15 min, 3,000 × *g*, 4°C). The aqueous phase (~11 mL) was collected, 0.5 g/mL of cesium chloride was added, and the suspension was layered onto a discontinuous cesium chloride gradient as previously described (38).

Two samples of filter-sterilized high-titer JS1 stocks originating from two distinct plaques were purified by cesium chloride gradient centrifugation. The two gradient-purified samples were visualized by TEM six times, on different days, each independently prepared for negative stain TEM to be certain that the TEM samples were representative and reproducible.

### Mass spectrometry of phage virion proteins

For proteomic analysis, CsCl-purified phage suspension was digested with trypsin and processed for mass spectrometry. Six microliters of the suspension (0.04 µg/µL) was injected into an Orbitrap Exploris 480 (Thermo Scientific) mass spectrometer equipped with a Dionex Ultimate 3000 RSLCnano LC system and a high-field asymmetric waveform ion mobility spectrometer (FAIMS) unit. Peptides were trapped on an Acclaim PepMap 100 trap column (5 μm, 100 μm × 2 cm, 100Å, nanoViper; Thermo Scientific) and separated on an Acclaim PepMap RSLC analytical column (2 μm, 75 μm × 50 cm, 100Å, nanoViper; Thermo Scientific). The injection volume was fixed at 2 µL. Samples were analyzed in a positive mode in data-dependent acquisition (DDA), coupled with high-field asymmetric waveform ion mobility spectrometer (FAIMS) using label-free quantification (LFQ). Carbamidomethylation of cysteines was set as fixed modification, and oxidation of methionines and acetylation of the N-terminus were set as variable modifications. Peptides and protein identifications were performed on Fragpipe v22.0 (162) with MSfragger v4.1 (163) search engine and were identified with a predefined false discovery rate (FDR) threshold of 1% using the phage and its host genome as database.

### Phage host range and efficiency of killing

A total of 44 *Staphylococcus* strains representing seven different species (Table S1) were used for the host range of JS1, Biyabeda-mokiny 1 (BB1), Biyabeda-mokiny 2 (BB2), and Koomba-kaat 1 (KK1) phage. A 10-fold serial dilution of the normalized high-titer phage stocks (10^9^ PFU/mL) was prepared (from 10^−1^ to 10^−6^) using SM buffer. A volume of 100 μL of the overnight bacterial culture was added into 3 mL of soft agar (0.3% TSA), mixed, and spread on the bottom agar plate (1.5% TSA) and allowed to solidify. Following the solidification of the soft agar, 2 µL of each dilution of the high-titer phage lysates were dispensed onto each Petri plate and incubated for 18–24 h at 37°C. In total, 2 µL of SM buffer was also spotted as a control. The area spotted with phage lysate and SM buffer was evaluated for lytic phage activity. The buffer did not produce any spot on any of the strains. The host range of each of the phages on all of the strains was determined by a similar scoring system described previously (56). However, in our case, we recognized four categories for the scoring of spot-testing: spots that are off-scale in these dilutions for high-throughput testing and showed PFU/mL in plaque assays that were equivalent to or more active than the isolation host *S. xylosus* WT2481, these were denoted “dark blue.” Strains that gave spots that are on-scale, with apparent plaques visible at the highest dilution for high-throughput testing and were thus found to be 10–100 times less active in plaque assays than the isolation strain *S. xylosus* WT2481, these were denoted “medium blue.” A light blue color designation was given to strains that showed positive spot testing, but only small hazy plaques at low dilution in plaque assays. A “gray” designation represents strains where no positive spot test was detected. This semi-quantitative assessment of the scoring system used a volume of 10 µl of serial 10-fold dilutions (10^−1^–10^−12^) of normalized high-titer phage stock (10^12^ PFU/mL) was mixed with 100 µL of the overnight host culture, added into 3 mL of soft agar, mixed and spread on the bottom agar plate, and incubated for 18–24 h at 37°C for the formation of visible plaques.

### Expression and purification of putative hydrolase proteins

The open-reading frames corresponding to JS1_0031, JS1_0032, and JS1_0224 were amplified using Phusion High-Fidelity DNA Polymerase system (New England Biolabs) from phage JS1 cDNA using hydrolase-specific primers (Table S5). PCR products were cloned into the protein expression vector pPROEX htb (Thermo Fisher Scientific) with an N-terminal hexahistidine tag. The expression constructs were transformed by heat shock into either *E. coli* BL21 Star (DE3) cells or C41 (DE3) cells (Novagen). Transformants were grown in 1.25 L of Luria-Bertani broth (10 g Tryptone, 5 g yeast extract and 10 g NaCl per liter of water) at 37°C until they reached an optical density at 600 nm (OD_600_) of 0.8–1.2. The protein expression was then induced with 0.5 mM isopropyl-β-d-thiogalactopyranoside (IPTG, Astral Scientific) with shaking overnight at 18°C, and the cells were harvested by centrifugation (10 min, 5,000 × *g*, 4°C).

Protein purification was carried out as described previously (139, 164). The cells were lysed in lysis buffer (20 mM Tris pH 8.0, 200 mM NaCl, and 5 mM imidazole) using an Avestin Emulsiflex C3 cell press (three passes). Cell lysates were clarified by centrifugation (30 min, 27,000 × *g*, 4°C), and His-tagged proteins were purified from the supernatant via nickel affinity chromatography. Proteins were loaded onto a 5 mL nickel HisTrap HP column (Cytiva) and eluted using a gradient (5%–50%) of 20 mM Tris pH 8.0, 200 mM NaCl, and 1 mM imidazole. For JS1_0031 and JS1_0032, no elution peaks were observed, and assessment of the purification through Coomassie-stained reducing sodium dodecyl sulfate-polyacrylamide gel electrophoresis (SDS-PAGE) found the proteins to be insoluble following cell lysis and clarification. For JS1_0224, an elution profile showed peaks corresponding to the protein, and these peak fractions were dialyzed (Spectra/Por Dialysis Membrane MWCO 6–8 kDa) against the size exclusion chromatography (SEC) buffer (20 mM Tris pH 8.0 and 150 mM NaCl) to remove imidazole. The dialyzed material was then concentrated (Amicon Ultra Centrifugal Filter, MWCO 30 kDa) and further purified by SEC over a HiLoad 16/600 Superdex 200 pg column (Cytiva) equilibrated in the SEC buffer. Coomassie-stained reducing SDS-PAGE was used to confirm the purity of JS1_0224, and an excised band from the Coomassie-stained gel was analyzed by mass spectrometry to confirm it as JS1_0224. The fractions containing the purified protein were then concentrated (Amicon Ultra Centrifugal Filter, MWCO 30 kDa), the concentration of JS1_0224 was determined using a bicinchoninic acid (BCA) assay (Thermo Fisher Scientific), and aliquots of the concentrated JS1_0224 were flash frozen in liquid nitrogen for storage at −80°C.

### Imaging of phage virions by TEM and cryoelectron microscopy

Grids for TEM imaging were prepared by pipetting 7 µL of phage suspension onto custom-made carbon-stabilized ultrathin formvar-coated grids that had been treated with UV light (265 nm wavelength) for 30 min to enhance hydrophilicity (165). After allowing the phage to adsorb for 2 min, Whatman filter paper was used to blot excess fluid from grids. The grids were washed by incubating them in SM buffer for 2 min. The grids were then stained three times (30 s each for first and second time and 1 min for the third time) with 10 µL 1% uranyl acetate (pH 7.4) stain with blotting after each round of staining and were then left to dry for 30 min. The grids were examined with a TEM (JEM-1400; JEOL, Japan) at an accelerating voltage of 80 kV.

For cryo-electron microscopy, approximately 3 μL of phage suspension was applied to Quantifoil R1.2/1.3 Cu 200 mesh holey carbon grids (Quantifoil Micro Tools GmbH, Großlöbichau, Germany) under chamber conditions of 90% humidity and 25°C and vitrified using a Leica EM GP2 Automatic Plunge Freezer (Leica Microsystems, Wetzlar, Germany). Data were collected on an FEI Titan Krios TEM operated at an accelerating voltage of 300 kV. Images were contrast-transfer function (CTF)-corrected using cryoSPARC software package for display. No structural reconstruction was performed in this study.

### Imaging of phage/phage-hydrolase-treated host by scanning electron microscopy

For visualization of the phage and host interaction or the effect of phage hydrolase on host by scanning electron microscopy (SEM), a monolayer was prepared by placing 20 μL of phage host suspension (log phase cells washed with PBS) onto a fresh gold-coated (∼20 nm) silicon wafer (5 × 5 mm). After settling the cells for about 5 min, they were washed with PBS (166, 167), followed by the addition of 20 μL of either the phage suspension for 5, 10, 20, and 40 min separately or the purified hydrolase for 5, 10, 15, 20, 25, and 30 min (37°C) separately and washing with PBS (166). The wafer was then placed in 2.5% glutaraldehyde in PBS solution for 15 min, washed with deionized water, and dehydrated by immersing in increasing concentrations (30%, 50%, 70%, 90%, 95%, and 3 × 100%) of ethanol for 5 min each. Residual ethanol was removed with a critical point dryer EM CPD300 (Leica). The sample was mounted on a standard metal SEM stub and coated with a ∼10 nm thick gold layer using a sputter coater EM ACE600 (Leica). The samples were examined within the FEG-SEM ThermoFisher Elstar G4 at an accelerating voltage of 2 kV, secondary electron mode, and a working distance of 2 mm, operating in immersion mode with the through lens detector. Best resolution was obtained using a monochromated 6.3 pA beam probe and dwell time 10 μs/pixel (166–168). A large area was collected automatically using ThermoFisher MAPS software (images not shown).

### FIB-SEM visualization of the cell wall

A monolayer of cells was prepared on gold-coated sapphire disks (6 mm, M. Wohlwend GmbH, art. 616), the same way as it is described above for the SEM preparation. After a brief washing in PBS, the disc was placed on the flat side of 6 mm aluminum carrier (M. Wohlwend GmbH, art. 1320); then, a hexadecene-coated 6-mm aluminum carrier (50 µm cavity, M. Wohlwend GmbH, art. 797) was placed above, and the “sandwich” was frozen using a high-pressure freezing machine HPF Compact 03 (M. Wohlwend GmbH). After freezing, the samples were processed using an automatic freeze-substitution unit AFS2 (Leica, Austria). The freeze substitution was conducted in 2% OsO_4_ acetone solution for 48 h at – 90°C, then increasing temperature (4 deg/h) to 15°C. After freeze substitution, the discs were washed in acetone and embedded in Epon resin (168). The FIB-SEM data sets were collected using a Helios 5 Hydra DualBeam cryo-plasma-FIB-SEM (ThermoFisher).

### SYBR Green-based thermal melting analysis of JS1 

Thermal melting analysis was performed to evaluate SYBR Green I fluorescence behavior in the presence of phage DNA release. SYBR Green I Nucleic Acid Gel Stain (10,000× in DMSO, Thermo Fisher Scientific) was diluted in SM buffer to working concentrations prior to use. CsCl-purified JS1 phage DNA was mixed with the dye and subjected to a thermal ramp from 28°C to 95°C, increasing by 1°C per step. Each step included a defined hold time to allow signal stabilization. Fluorescence was monitored in the green channel (510 nm emission), and all samples were analyzed in duplicate. SM buffer alone served as a negative control.

### Size measurement and curvature analysis of phage virions

The size measurements of bacteriophages were performed using the "Digital Micrograph Software (DM3)" within the Gatan Microscopy Suite (169). Samples were prepared and imaged using a TEM, and the resulting micrographs were analyzed using DM3 software. Fifteen individual phages were measured to determine their head diameter, tail length, and tail width. Measurements were used to calculate the mean and standard deviation (SD), statistically assessing the phage dimensions. Curvature of phage tails was analyzed using the Kappa plugin (57) within the ImageJ (Fiji) (170) software package. The Kappa plugin fits a curve to the selected phage tail and calculates key parameters, including average curvature and curvature standard deviation, offering a detailed assessment of the tail’s bending characteristics.

### Structural predictions and comparisons using AlphaFold3

The structure predictions of the JS1_0021 TTP assembly were performed using AlphaFold3 (59), and analysis and depiction of structures were performed using UCSF ChimeraX (v 1.8) (171). The structure predictions of JS1_0031, JS1_0032, and JS1_0224 were performed using AlphaFold, and analysis and depiction of structures were performed using UCSF ChimeraX (v 1.10) (171).

### Phage host range and efficiency of killing

A total of 44 *Staphylococcus* strains representing six different species (Table S1) were used for the host range of JS1, Biyabeda-mokiny 1 (BB1), Biyabeda-mokiny 2 (BB2), and Koomba-kaat 1 (KK1) phage. A 10-fold serial dilution of the normalized high-titer phage stocks (10^12^ PFU/mL) was prepared (from 10^−1^ to 10^−6^) using SM buffer. A volume of 100 μL of the overnight bacterial culture was added into 3 mL of soft agar (0.3% TSA), mixed, and spread on the bottom agar plate (1.5% TSA), and allowed to solidify. Following the solidification of the soft agar, 2 µL of each dilution of the high-titer phage lysates were dispensed onto each Petri plate and incubated for 18–24 h at 37°C. The area spotted with phage lysate was evaluated for lytic phage activity. The host range of each of the phages on all of the strains was determined by a similar scoring system described previously (56). However, in our case, we recognized four categories for the scoring of spot-testing: spots that are off-scale in these dilutions for high-throughput testing and showed PFU/mL in plaque assays that were equivalent to or more active than the isolation host *S. xylosus* WT2481, these were denoted “blue.” Strains that gave spots that are on-scale, with apparent plaques visible at the highest dilution for high-throughput testing and were thus found to be 10–100 times less active in plaque assays than the isolation strain *S. xylosus* WT2481, these were denoted “red.” A “yellow” color designation was given to strains that showed positive spot testing but only small hazy plaques at low dilution in plaque assays. A “gray” designation represents strains where no positive spot test was detected. This semi-quantitative assessment of the scoring system used a volume of 10 µl of serial 10-fold dilutions (10^−1^– 10^−12^) of normalized high-titer phage stock (10^12^ PFU/mL) was mixed with 100 µL of the overnight host culture, added into 3 mL of soft agar, mixed and spread on the bottom agar plate, and incubated for 18–24 h at 37°C for the formation of visible plaques.

## Data Availability

The genome sequence of the phage JS1 has been deposited with accession number PQ849840, and the genome sequences of the 16 *Staphylococcus* strains isolated in this study have been deposited with accession numbers listed in Table 1.

## SUPPLEMENTAL MATERIAL

Supplemental material mbio.03829-25-s0001.pdf Figures S1-S5 and Tables S1, S4, and S5.

Table S2 mbio.03829-25-s0002.xls Metadata of *Staphylococcus* core genomes.

Table S3 mbio.03829-25-s0003.xls Annotation of *Staphylococcus* phage JS1 genome.

ASM does not own the copyrights to Supplemental Material that may be linked to, or accessed through, an article. The authors have granted ASM a non-exclusive, world-wide license to publish the Supplemental Material files. Please contact the corresponding author directly for reuse.
