# Supplementary material for: Staphylococcus species infected by a bacteriophage with a tail that is both curved and contractile
Source: mBio. 2026 Feb 6;17(3):e03829-25. doi: 10.1128/mbio.03829-25 (PMC12977511; doi:10.1128/mbio.03829-25)
Supplement: Supplemental material — Figures S1-S5 and Tables S1, S4, and S5. [file mbio.03829-25-s0001.pdf]

Supplementary Materials for

***Staphylococcus* species infected by a bacteriophage with a tail that is both flexible and contractile.**

Suhani et al.

Corresponding author: Trevor Lithgow, trevor.lithgow@monash.edu

The PDF file includes:

Figures S1 to S5

Tables S1, S4 and S5

Other Supplementary Material for this manuscript includes the following:

Tables S2 and S3 (separately attached as excel file)

Supplementary Figure S1. Comparison of phage JS1 with related phages. (A) Phylogenetic tree of JS1 with related genomes. The circular proteomic tree of ViPTree analysis with 496 phage genomes using a protein distance metric based on normalized tBLASTx scores. The four Herelleviridae phages used in this study highlighted with sky blue, red, navy blue and magenta stars and labelled with arrows. **(B)** Genome-BLAST distance phylogeny (GBDP) of phage JS1 and 25 other Herelleviridae phages by VICTOR (see Methods). The phylogenomic GBDP tree inferred using the formulas D4, yielding average support of 40%. The numbers above branches are GBDP pseudo-bootstrap support values from 100 replications. The branch lengths of the resulting VICTOR trees are scaled in terms of the respective distance formula used. The phages clustered into groups defined by distinct Genus (4 groups). Annotated alongside the tree are the isolation host species (Host) and physical sourcing (Source). **(C)** Genome sequence comparison between *Staphylococcus* phages JS1 and vB_Sau_Clo6 using clinker[172]. The predicted genes are color coded by the predicted function of the proteins that they encode as indicated in the legend. Homologous regions in the two genomes detected by global alignment are connected by segments colored in shades of grey based on amino acid identity: black for identical, through to white being no detectable identity.

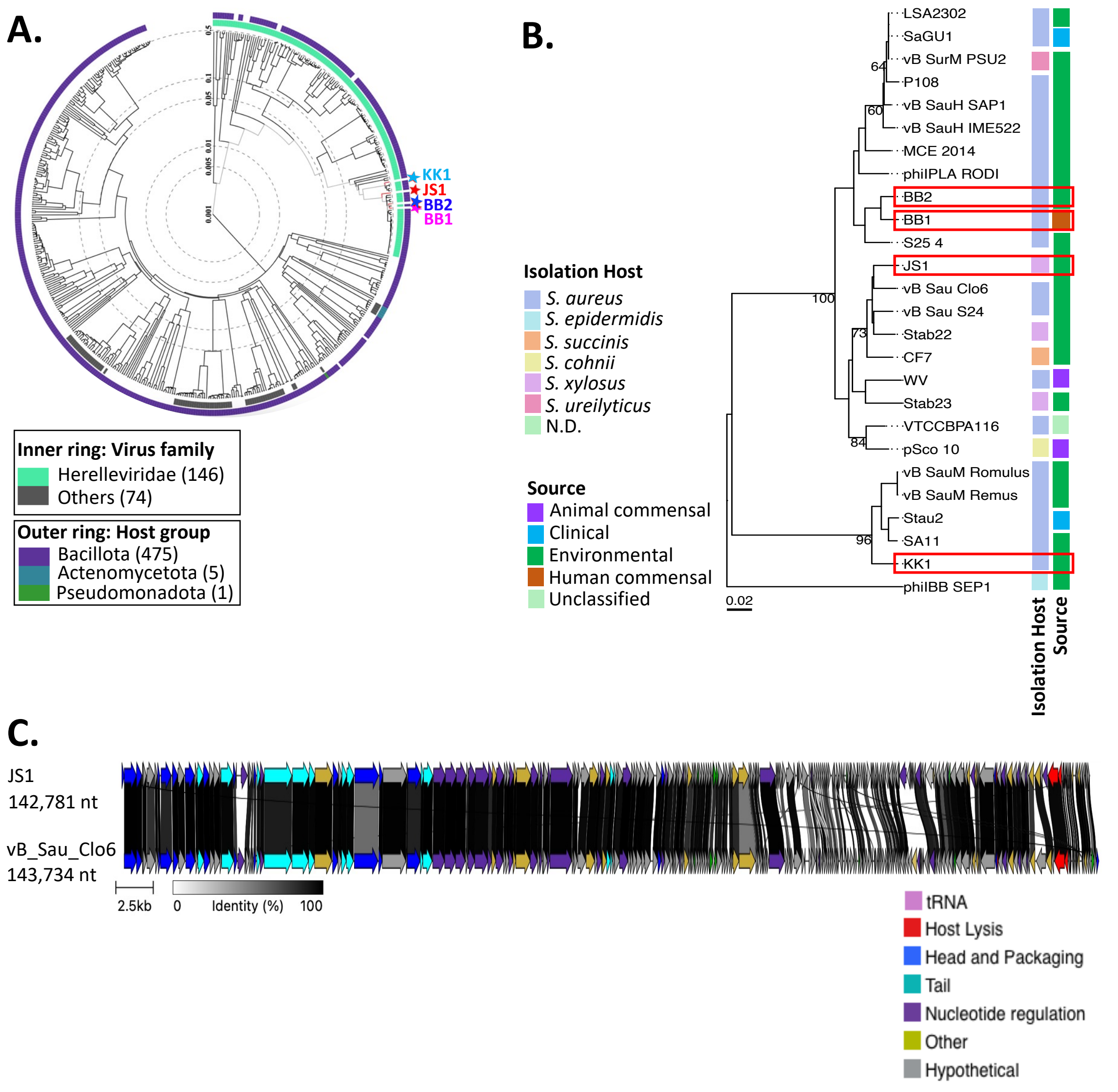

Supplementary Figure S1

Supplementary Figure S2. Conserved domains and structural prediction of hydrolases. Alphafold was used to predict structures for proteins designated as hydrolases (details in main text). **(A)** The color-coding of the structure of JS1_0031 represents the confidence of the prediction, where blue = high confidence prediction. The the PAE box-plot representing confidence regions corresponding to domain boundaries is shown, as is the Per-residue confidence score (pLDDT) for the five top-ranked AlphaFold structure predictions. These confidence regions correspond to domain boundaries, corresponding to the HMM-based predictions for conserved domains shown in the linear representation of the protein from the N-terminus to the C-terminus. Below: the corresponding Alphafold predicted structure has also been coloured from N-terminus (blue) to C-terminus (red) to assist with orientation when considering these conserved domains. **(B)** Equivalent predictions for JS1_0032 structure having an N-terminal SH3bC-terminal cell-binding domain and C-terminal NlpC domain, characteristic of a family of NlpC/P60 family of characterised bacterial cell wall hydrolases **(C)** Equivalent predictions for JS1_0224 showed it to be related to phage endolysins such as LysK, with a predicted modular structure with two predicted catalytic domains: a CHAP domain and a central amidase-2 (*N*-acetylmuramoyl-l-alanine amidase) domain, completed with a C-terminal SH3b cell-binding domain[54].

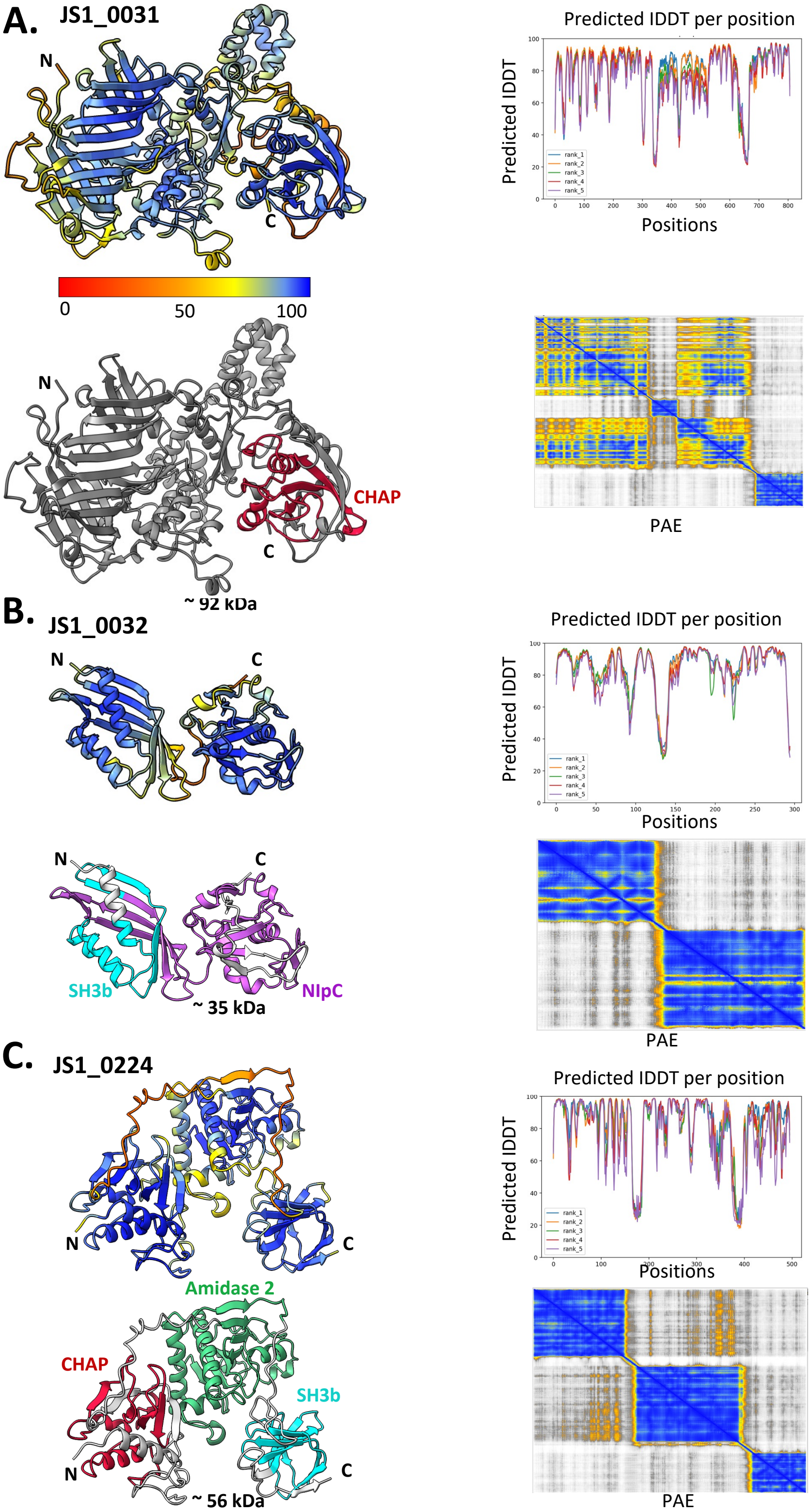

Supplementary Figure S2

Supplementary Figure S3. Primary data for host-range determination for phages JS1, BB1, BB2 and KK1. The host-range for the phages JS1, BB1, BB2 or KK1 (summarized in **Fig. 5D**) was quantified, representative plates of some of the indicated Staphylococcal strains tested are shown. The plates were selected to highlight cases of low, high and very high titer plaque formation for phage JS1. From these plates and the plating of dilutions of the phage stock activity in PFU/mL was calculated. Insets (in squares) show higher magnification of a section of the plate. Scoring of the plates is as summarized in Figure 5: blue squares denote strains where JS1 activity was observed to be equal or better PFU/mL than the isolation host *S. xylosus* WT2481 in plaque assays. The red squares represent plates equating to 10-100 fold less PFU/mL than the isolation host *S. xylosus* WT2481, yellow squares show some small hazy plaques at lower dilutions (as well as some clearance on plates with spot tests, Figure 5). In addition, two plates representative of strains where phage JS1 had no activity in spot tests and no plaques visible at any dilution are shown (*S. lentus* PT1483, *S. aureus* SA75).

Supplementary Figure S4. Virion morphology revealed by electron microscopy for phage JS1. Sample views of JS1 virions. From across this sampling, selections were made of 18 of the most curved virions and the curvature was analysed and quantified using Kappa software[56] (see Methods; **Fig. 4**).

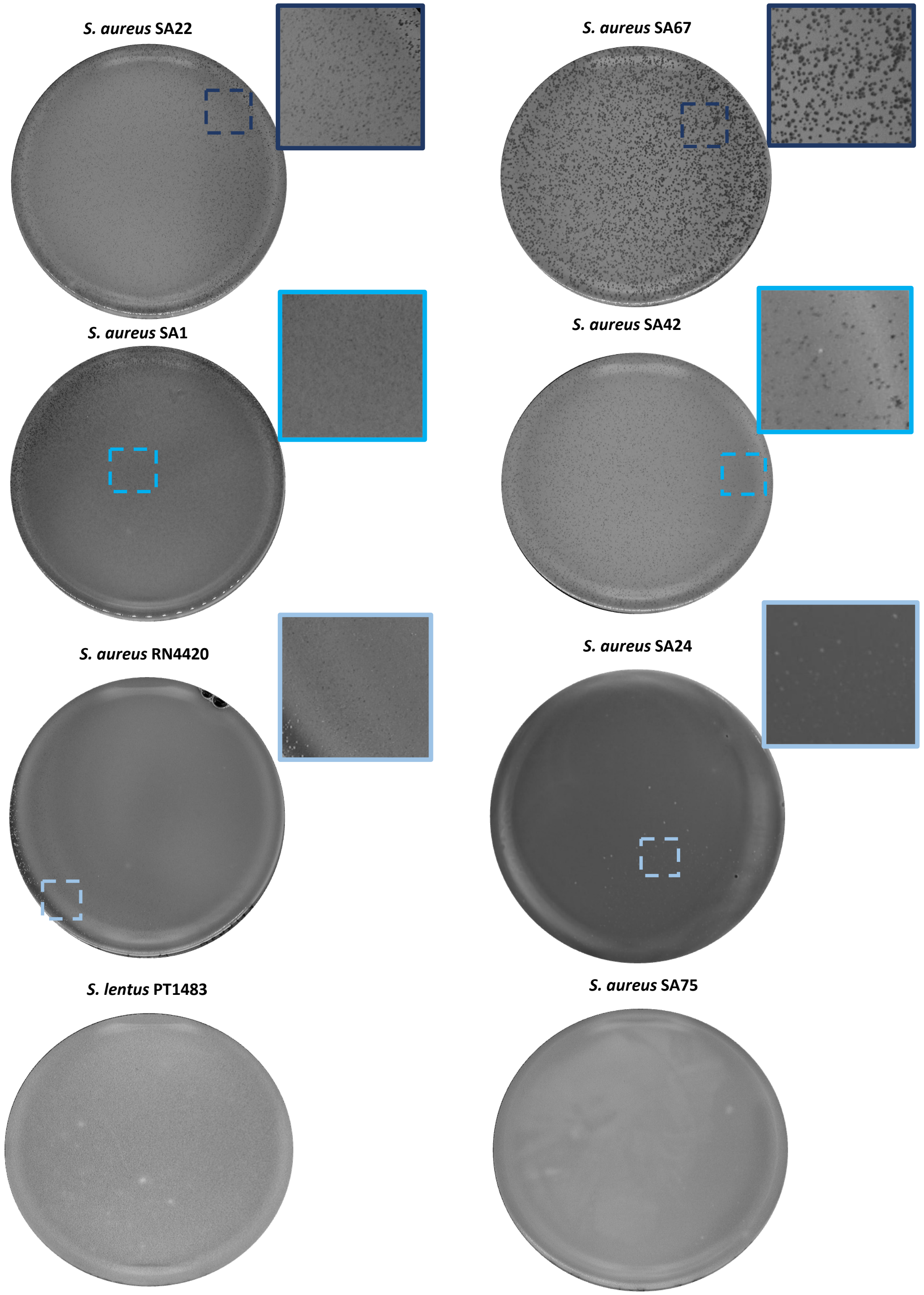

Supplementary Figure S3

Supplementary Figure S4. Virion morphology revealed by electron microscopy for phage JS1. Sample views of JS1 virions. From across this sampling, selections were made of 18 of the most curved virions and the curvature was analysed and quantified using Kappa software[56] (see Methods; **Fig. 4**).

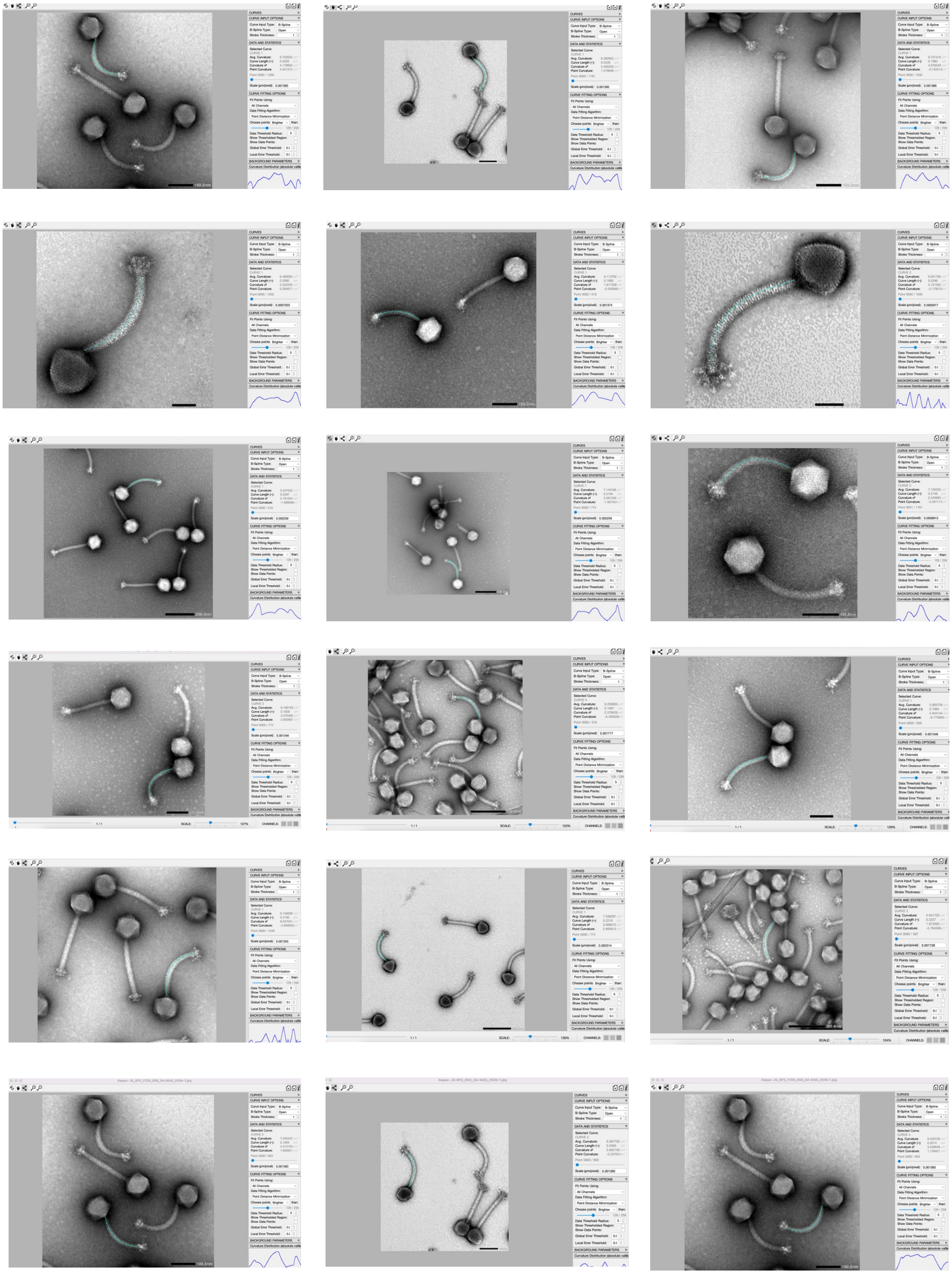

Supplementary Figure S4

Supplementary Figure S5. Electron micrographs. In the interest of clarity, the brightness and contrast were adjusted on three micrographs shown in this paper. Here, the original micrograph is shown alongside the version where the brightness and contrast of the images were adjusted. **(A)** as shown in Figure 2F. **(B)** as shown in Figure 3C. **(C)** as shown in Figure 6A.

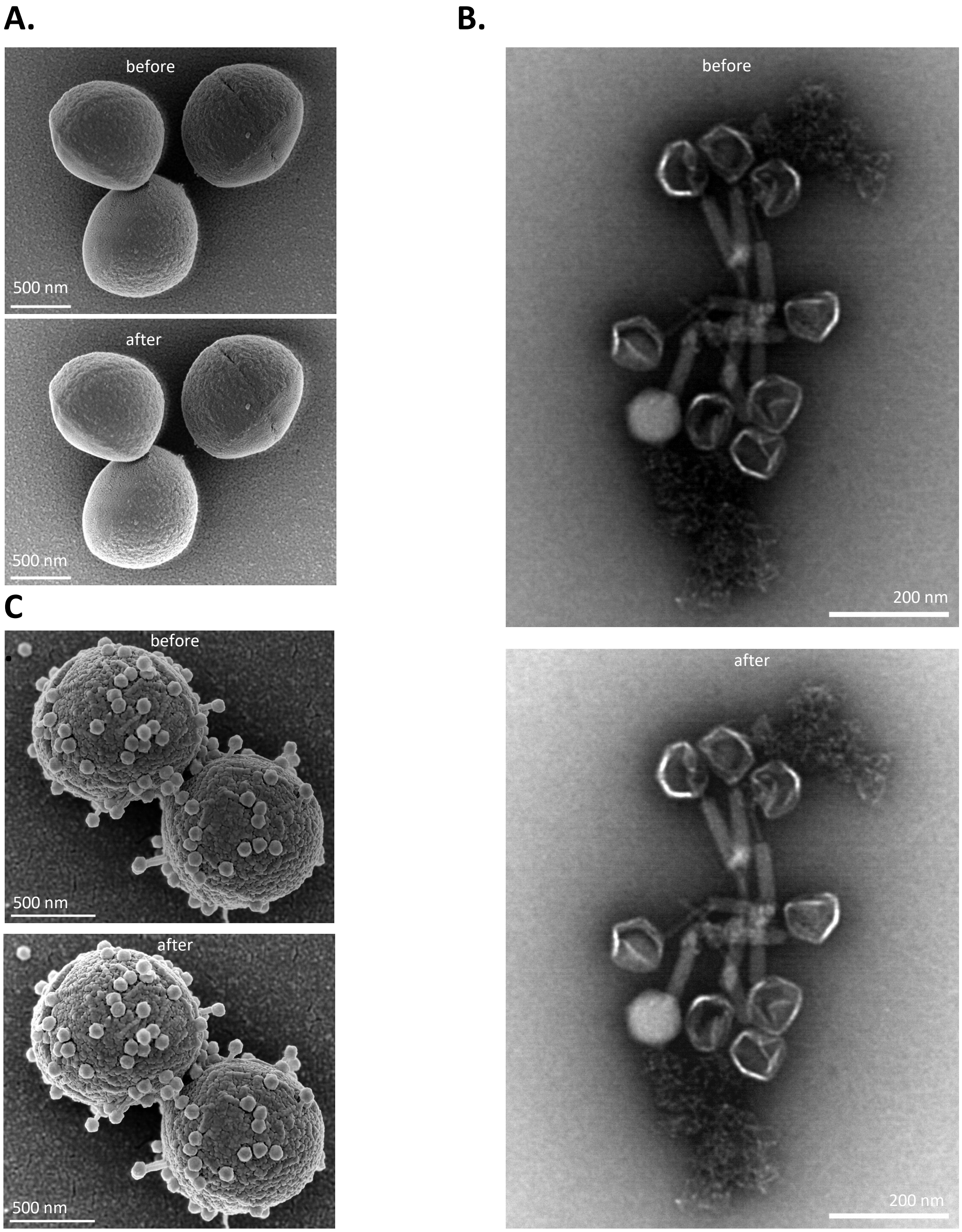

Supplementary Figure S5

Supplementary Table S1. Bacterial strains used in this study

Bacterial Strains	Source	Reference
S. aureus SA1	B2* - Non CF**	A. Kicic
S. aureus SA4	CF cough	A. Kicic
S. aureus SA5	CF cough	A. Kicic
S. aureus SA6	CF cough	A. Kicic
S. aureus SA9	ACFBAL***	A. Kicic
S. aureus SA20	Foot wound	A. Kicic
S. aureus SA24	Blood	A. Kicic
S. aureus SA25	Blood	A. Kicic
S. aureus SA26	Blood	A. Kicic
S. aureus SA76	CF airways	A. Kicic
S. aureus SA91	CF airways	A. Kicic
S. aureus MJB1	Lab strain	Belousoff et al[181]
S. aureus SA10	CF cough	A. Kicic
S. aureus SA11	CF cough	A. Kicic
S. aureus SA12	CF cough	A. Kicic
S. aureus SA19	CF cough	A. Kicic
S. aureus SA21	Wound	A. Kicic
S. aureus SA22	Shoulder fluid	A. Kicic
S. aureus SA40	Blood	A. Kicic
S. aureus SA41	Blood	A. Kicic
S. aureus SA42	Blood	A. Kicic
S. aureus SA66	Synovial Fluid, Knee	A. Kicic
S. aureus SA67	Endotracheal Aspirate	A. Kicic
S. aureus SA68	Abscess Fluid, Neck	A. Kicic
S. aureus SA72	Bronchoalveolar Washing	A. Kicic
S. aureus SA75	Bronchoalveolar Washing	A. Kicic
S. *aureus* ATCC6538	Lab strain	ATCC[182]
S. aureus RN4420	Lab strain	Nair et al[183]
S. cohnii WT1082	wastewater	This study
S. equorum PT0982	wastewater	This study
S. equorum PT0983	wastewater	This study
S. lentus PT1482	wastewater	This study
S. lentus PT1483	wastewater	This study
S. lentus PT1484	wastewater	This study
S. nepalensis WT3182	wastewater	This study
S. sciuri PT2983	wastewater	This study
S. sciuri WT1781	wastewater	This study
S. sciuri WT2482	wastewater	This study
S. sciuri PT0981	wastewater	This study
S. shinii ET3081	wastewater	This study
S. shinii WT3181	wastewater	This study
S. xylosus PT0984	wastewater	This study
S. xylosus WT2481	wastewater	This study
S. xylosus ET2381	wastewater	This study

*B2- β2 agonists
** CF- Cystic Fibrosis
*** ACFBAL- Australasian CF Bronchoalveolar Lavage

Supplementary Table S4. Summary information on phage JS1 and closely related phages

Staphylococcus Phage	NCBI Accession	Genome Length	Isolation host	Genus	Source	Morphotype
JS1	PQ849840	142781	*S. xylosus*	Unclassified	Sewage	Myo-like
phiIBB-SEP1	NC_041928	139928	*S. epidermidis*	*Sepunavirus*	Sewage	Myo-like
vB_SauM_Romulus	NC_020877	131332	*S. aureus*	*Silviavirus*	Sewage	Myo-like
vB_SauM_Remus	NC_022090	134643	*S. aureus*	*Silviavirus*	Sewage	Myo-like
Stau2	NC_030933	133798	*S. aureus*	*Silviavirus*	Clinical	Myo-like
SA11	NC_019511	136326	*S. aureus*	*Silviavirus*	Sewage	Sipho-like
phiIPLA-RODI	NC_028765	142348	*S. aureus*	*Kayvirus*	Sewage	Myo-like
MCE-2014	NC_025416	141907	*S. aureus*	*Kayvirus*	Sewage	Myo-like
S25-4	NC_022918	132123	*S. aureus*	*Kayvirus*	Sewage	Myo-like
P108	NC_025426	140807	*S. aureus*	*Kayvirus*	Sewage	Myo-like
vB_Sau_Clo6	KY794642	143734	*S. aureus*	Unclassified	Sewage	Myo-like
vB_Sau_S24	KY794643	139997	*S. aureus*	Unclassified	Soil	Myo-like
CF7	PP034388	138217	*S. succinis*	Unclassified	Sewage	Myo-like
Stab22	LR215721	154499	*S. xylosus*	Unclassified	Sewage & river water	Myo-like
Stab23	LR215720	155962	*S. xylosus*	Unclassified	Sewage & river water	Myo-like
vB_SauH_IME522	MN304941	140246	*S. aureus*	*Kayvirus*	Sewage	Myo-like
vB_SauH_SAP1	MT786458	143375	*S. aureus*	*Kayvirus*	Sewage	Myo-like
LSA2302	PP140390	141325	*S. aureus*	*Kayvirus*	Sewage	Myo-like
SaGU1	LC574321	140909	*S. aureus*	*Kayvirus*	Clinical	Myo-like
vB_SurM-PSU2	OP913462	142537	*S. ureilyticus*	*Kayvirus*	Sewage	Myo-like
HSA30	MG557618	140358	*S. aureus*	*Kayvirus*	Unclassified	Myo-like
WV	MT787017	141342	*S. aureus*	Unclassified	Slaughterhouse	Myo-like

VTCCBPA116	MN935200	110012	*S. aureus*	Unclassified	Unclassified	Unclassified
pSco-10	KX011028	101986	*S. cohnii*	Unclassified	Duck faeces	Myo-like
Biyabeda-mokiny 1 (BB1)	OP263967	141091	*S. aureus*	*Kayvirus*	Breastmilk	Myo-like
Biyabeda-mokiny 2 (BB2)	OP263968	140800	*S. aureus*	*Kayvirus*	Sewage	Myo-like
Koomba-kaat 1 (KK1)	OP263969	135469	*S. aureus*	*Silviavirus*	Sewage	Myo-like

Supplementary Table S5: List of primers used to amplify putative JS1 hydrolase proteins.

**Primer Name**	**Primer Sequence** (underline: restriction enzyme cut sites)	**Cloning restriction enzymes**
JS1_0031_F<br>JS1_0031_R	5'-GCGCCATGGGCATGCGTAGAATAAGAAGACCTAAGGTAAG-3'<br>5'-GCCAAGCTTTTATTTCTTATCATAAATGAATTGAGCTTC-3'	NcoI<br>HindIII
JS1_0032_F<br>JS1_0032_R	5'-CGCCATGGGCATGGCAACAG-3'<br>5'-GCCAAGCTTGCTTAAATATACACCTC-3'	NcoI<br>HindIII
JS1_0224_F<br>JS1_0224_F	5'-GCGCCATGGGCATGGCTAAGACTCAAGCAG-3'<br>5'-GCCAAGCTTTTAACTCTTGAATGTCCCCCAGGC-3'	NcoI<br>HindIII
